# Growth Factor-Activated Stem Cell Circuits and Stromal Signals Cooperatively Accelerate Non-Integrated iPSC Reprogramming of Human Myeloid Progenitors

**DOI:** 10.1371/journal.pone.0042838

**Published:** 2012-08-08

**Authors:** Tea Soon Park, Jeffrey S. Huo, Ann Peters, C. Conover Talbot, Karan Verma, Ludovic Zimmerlin, Ian M. Kaplan, Elias T. Zambidis

**Affiliations:** 1 Institute for Cell Engineering, School of Medicine, Johns Hopkins University, Baltimore, Maryland, United States of America; 2 Division of Pediatric Oncology, Sidney Kimmel Comprehensive Cancer Center at Johns Hopkins, Baltimore, Maryland, United States of America; 3 Institute for Basic Biomedical Sciences at Johns Hopkins, Baltimore, Maryland, United States of America; University of Minnesota Medical School, United States of America

## Abstract

Nonviral conversion of skin or blood cells into clinically useful human induced pluripotent stem cells (hiPSC) occurs in only rare fractions (∼0.001%–0.5%) of donor cells transfected with non-integrating reprogramming factors. Pluripotency induction of developmentally immature stem-progenitors is generally more efficient than differentiated somatic cell targets. However, the nature of augmented progenitor reprogramming remains obscure, and its potential has not been fully explored for improving the extremely slow pace of non-integrated reprogramming. Here, we report highly optimized four-factor reprogramming of lineage-committed cord blood (CB) myeloid progenitors with bulk efficiencies of ∼50% in purified episome-expressing cells. Lineage-committed CD33^+^CD45^+^CD34^−^ myeloid cells and not primitive hematopoietic stem-progenitors were the main targets of a rapid and nearly complete non-integrated reprogramming. The efficient conversion of mature myeloid populations into NANOG^+^TRA-1-81^+^ hiPSC was mediated by synergies between hematopoietic growth factor (GF), stromal activation signals, and episomal Yamanaka factor expression. Using a modular bioinformatics approach, we demonstrated that efficient myeloid reprogramming correlated not to increased proliferation or endogenous Core factor expressions, but to poised expression of GF-activated transcriptional circuits that commonly regulate plasticity in both hematopoietic progenitors and embryonic stem cells (ESC). Factor-driven conversion of myeloid progenitors to a high-fidelity pluripotent state was further accelerated by soluble and contact-dependent stromal signals that included an implied and unexpected role for Toll receptor-NFκB signaling. These data provide a paradigm for understanding the augmented reprogramming capacity of somatic progenitors, and reveal that efficient induced pluripotency in other cell types may also require extrinsic activation of a molecular framework that commonly regulates self-renewal and differentiation in both hematopoietic progenitors and ESC.

## Introduction

Although the derivation of human induced pluripotent stem cells (hiPSC) via ectopic expression of defined transcription factors holds great potential for regenerative medicine and disease modeling, factor-driven reprogramming of human somatic cells is slow, inefficient, and produces highly variable qualities of pluripotency. This inefficiency stems from the fact that defined transcription factors trigger obscure epigenetic events that result in a stable pluripotent state in only a rare fraction of transgene-expressing somatic cells. More refined, nonviral non-integrating reprogramming methods are expected to produce hiPSC lines with fewer epigenomic aberrations, and may ultimately be more suitable for therapeutic applications. However, non-integrated reprogramming of human somatic fibroblasts [1]–[3] or stem-progenitors is even less efficient (∼0.001–0.5% of input cells) and more technically challenging than with viral constructs [4]–[6]. This inefficiency stems from an inherently low nonviral gene transfer efficiency of human cells which often requires augmentation with chromatin-modifying small molecules, or multiple factor transfections of the same dividing and expanded target populations [2] for reliable derivation of hiPSC clones.

Recent evidence suggests that all proliferating somatic cells likely have the capacity to become reprogrammed to a pluripotent state following sustained ectopic expression of defined factors, albeit with long latency periods [7]. However, the factor-driven somatic activation of transcriptional networks that initiates and maintains the induced pluripotent state is regulated by both cell intrinsic and extrinsic micro-environmental factors [8]. The intrinsic factors that determine the rate and efficiency of somatic cell reprogramming include the lineage type, developmental maturity, and chromatin state of the donor cell [9]–[11]. For example, reprogramming of developmentally immature neural [9], [10] and hematopoietic [12] stem-progenitors requires fewer defined factors (*e.g.,* only SOX2 and OCT4) than fully differentiated fibroblasts. The mechanism behind augmented progenitor reprogramming efficiency remains obscure, but has been suggested to be related to high endogenous expression of key reprogramming factors (*e.g.,* SOX2, KLF4) or an embryonic stem cell (ESC)-like epigenome that facilitate ectopic factor-driven reprogramming [6], [9]–[12]. However, despite the requirement of fewer defined factors, the human reprogramming efficiency of neural or hematopoietic stem-progenitors with one to seven factors has not been reported to be significantly higher than other more differentiated human donor cell types (∼0.001–0.5%) [4]–[6]. In contrast, an inducible transgenic mouse system that homogenously expressed the Yamanaka factors in all somatic donor cells reported that hematopoietic stem and progenitor cells generated murine iPSC with unprecedented efficiencies of 8–28% [11]. These high murine iPSC efficiencies suggest not only that hematopoietic progenitors may represent a uniquely amenable somatic donor type, but also that reprogramming efficiency for human hematopoietic progenitors has not been fully optimized. Such optimization may require not only improved gene transfer of defined factors into hematopoietic cells, but also identification of uniquely reprogrammable cellular phenotypes from mixed progenitor populations or additional unknown micro-environmental signals. Importantly, although hematopoietic stem-progenitors endogenously express multiple factors that may favor factor-driven chromatin remodeling [13]–[17], the notion that it is stem-progenitors and not more differentiated, lineage-committed cells in mixed progenitor populations that are the actual targets of facile reprogramming [6], [9]–[12] has not been fully explored.

Overcoming the barriers that prevent rapid and high-fidelity hiPSC generation ultimately requires an understanding of the regulatory networks that must be activated by defined factors to convert somatic cells to a high-fidelity state of pluripotency. Signals that enhance cell division accelerate these genetic and epigenetic cascades (*e.g*. ectopic LIN28 expression, or inhibition of the p53/p21 pathway) [7]. The molecular events that are triggered by ectopic factor expression are understood to be sequential, and regulated by distinct transcriptional networks that operate in the context of an ‘open’ chromatin state [18]. The events these networks orchestrate involve dynamic changes in somatic DNA methylation, histone modification, and nucleosome spacing [19], [20], and require the infrastructure of an OCT4 transcription factor-centered protein network [21]–[24]. An independent MYC complex-regulated network initiates the sequence of factor-driven reprogramming events by upregulating an embryonic metabolic program, and remodeling somatic heterochromatin to a less condense, more transcriptionally-permissive ESC-like state [25]–[27]. These primary events are subsequently followed by activation of the auto-regulatory Core circuit (*i.e.,* the SOX2-OCT4-NANOG (SON) network) [28]–[30] which initiates and maintains the pluripotent state, and also cooperates with Polycomb group (PcG) repressive complexes (*i.e*., PRC1, PRC2) to silence lineage specification [31]–[34]. These three major regulatory networks (active MYC complex targets, inactive PcG complex (PRC1, PRC2) targets, and active SON Core factor networks) represent independent and functionally separable transcriptional modules [27]. In cooperation with protein complexes that enzymatically regulate chromatin remodeling [21]–[24], [35]–[38], these transcriptional modules maintain and regulate the pluripotent state in both ESC and iPSC. The sequential epigenetic activation of these networks to ESC-like states by ectopic expression of defined factors represents the barrier to efficient factor-driven reprogramming.

Since hematopoietic cells represent a highly accessible cell type for generating clinically relevant pluripotent stem cells, we focused in these studies on unlocking their optimized reprogramming potential. We tested the hypothesis that the epigenetically malleable genome of hematopoietic stem-progenitors [13]–[17] can be fully activated by extrinsic signals from their micro-environmental niche [39]–[41]. We demonstrate that bone marrow stromal cell (BMSC) signals synergized with hematopoietic growth factors (GFs) to significantly accelerate the non-integrated reprogramming of human myeloid progenitors toward a high quality pluripotent state with strikingly rapid and near total completion. This high efficiency correlated not to endogenous expression of singular reprogramming factors (*e.g.,* SOX2, OCT4, NANOG) in myeloid progenitors, but to hematopoietic MYC, Polycomb, and OCT4 factor-associated networks that were revealed to be expressed at ESC-like levels in these progenitors following activation by hematopoietic GFs. The reconfiguration of these hematopoietic networks to hESC-like patterns was further accelerated by soluble and contact-dependent stromal signals, following a single episomal pulse of the four Yamanaka factors (SOX2, OCT4, KLF4, MYC).

This optimized experimental system facilitates the future elucidation of defined conditions for efficiently generating hiPSC from other cell types by focusing on the identification of common extrinsic factors and molecular networks that potentiate epigenetic plasticity and self-renewal in both somatic progenitors and pluripotent stem cells [42]. Moreover, the rapid and bulk generation of hiPSC en masse from myeloid populations provides a human reprogramming system for detailed kinetic biochemical, genetic, epigenomic, and proteomic investigations of synchronized populations.

## Results

### Brief Expansion of GF-activated CD34^+^ CB Cells on Irradiated BMSC Prior to Episomal Reprogramming Enhanced Hematopoietic Progenitor Frequency and Viability without Affecting Proliferation

**Figure 1:**
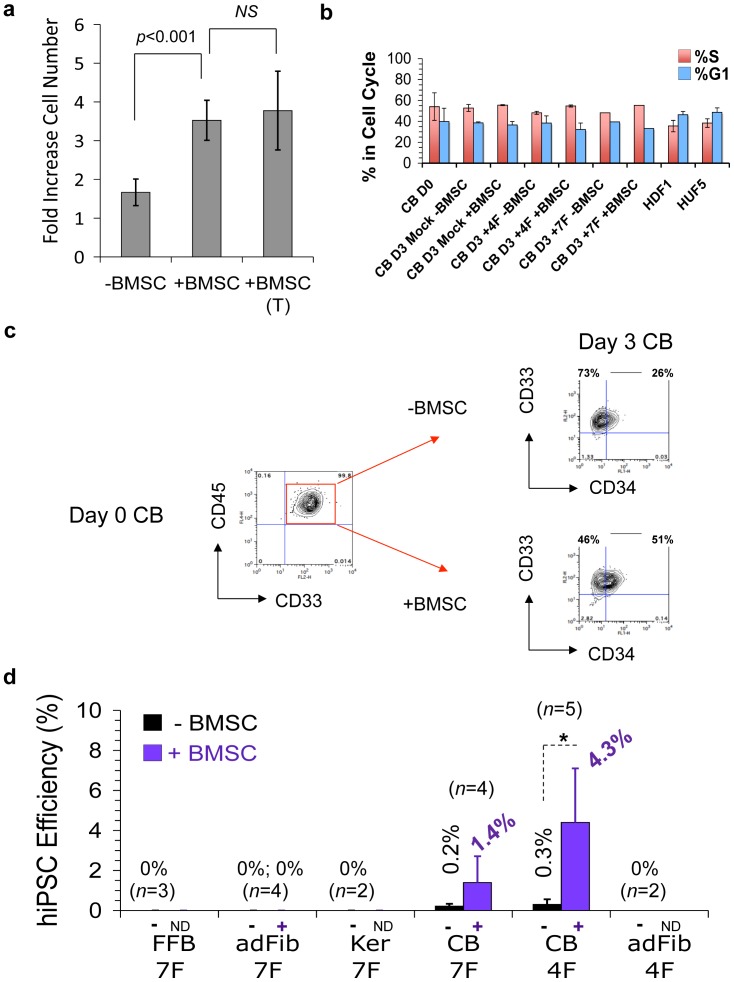
BMSC co-culture primed efficient non-integrated reprogramming of CB progenitors that required only four episomal Yamanaka factors on a single plasmid. The role of a brief 3-day BMSC co-culture of GF-activated CD34^+^ CB progenitors following a single pulse of episomal plasmids on Day 0, and the number of episomal factors required for efficient reprogramming. The experimental design is summarized in **Fig. S1**. (**a**) Soluble factors from brief BMSC co-culture preserved the viability of GF-activated CB progenitors. Viable CB cells were enumerated via Trypan Blue on Day 3 following nucleofection with four episomal factors (SOX2, OCT4, KLF4, MYC; 4F) on Day 0. Shown are the fold-increases of GF-treated CD34^+^ CB cells from Day 0 to Day 3 that resulted from: no BMSC co-culture (-BMSC), with irradiated BMSC co-culture (+BMSC), or with BMSC co-culture but separated by a Transwell insert (BMSC (T)). The Transwell culture insert prevented cell-cell contact between BMSC and CB cells, but allowed soluble stromal factors to diffuse freely to the cultured CB cells. Averages, SEM, and *p* values (Students t test) of *n* = 4 averaged experiments is shown. *NS* = not significant (*p*>0.05). (**b**) Brief co-culture of GF-activated CB cells with irradiated BMSC (+BMSC) did not increase the frequency of cells in G1 or S cell cycle phases at Day 3 (D3) of the reprogramming protocol (see **Fig. S1**) compared to GF alone (-BMSC). CB cells were nucleofected on Day 0 (D0) with 4F or 7F plasmids, or nucleofected with Amaxa buffer only (Mock). Cell cycle status of stromal-activated D3 CB, DO (pre-nucleofected) control CB cells, and adult fibroblasts (HDF1, HUF5; nucleofected with 7F) was determined on Day 3 of reprogramming protocols via FACS analysis of EdU incorporation and DNA content (7AAD), as described in Methods. Cells in G1 phase were defined as being EdU negative and 7AAD(2n); % cells in S phase were determined by gating on EdU positive 7AAD (2n+) populations. Data shown is the average of 2 experiments from individual batches of pooled CB donors run in triplicate. (**c**) GF-activated CB cells consist of a relatively homogenous myeloid population. By Day 3 of the reprogramming protocol (following +/− BMSC co-culture), both CD34^+^ and CD34^−^ GF-activated CD45^+^ CB progenitors had differentiated to primarily a myeloid CD33^+^ and CD13^+^ (not shown) phenotype. (**d**) Episomal reprogramming efficiencies of various somatic targets with either four factors (4F; SOKM, on a single plasmid, pEP4 EO2S EM2K) or seven factors (7F; SOKMNLT, on three separate plasmids), and with (+) or without (−) BMSC priming were determined by rapid AP staining (in triplicate) at 3 weeks following episomal nucleofections for CB progenitors (CB), fetal fibroblasts (FFB), adult fibroblasts (AdFib), and adult keratinocytes (Ker). Reprogramming efficiencies for emergence of Type III hiPSC [45] were quantitated in some experiments with parallel live TRA-1-81 staining, which gave similar results to enumeration of AP^+^ hESC-like colonies (**Fig. S3**). By 3 weeks post 4F nucleofections, the majority of AP^+^ hESC-like colonies had already converted with extremely high efficiencies to 50–80% fully reprogrammed SSEA4^+^TRA-1-81^+^NANOG^+^ Type III hiPSC (see also **Fig. 7a**). Each condition presented was repeated at least two to five times, as indicated, and with significance (t test; * = *p*<0.05) as indicated. 4F SOKM reprogramming of CB progenitors was even more robust than with equimolar DNA quantities of the 7F three-plasmid system (albeit initially less rapid). ND; BMSC co-culture was not done. With omission of the brief BMSC activation step, rarer (∼10–150-fold less) and slower-emerging CB-iPSC were produced with single-plasmid 4F nucleofections, but at frequencies that were still significantly greater than 7F reprogramming of fetal and adult fibroblasts, or adult keratinocytes.

We previously reported the derivation of nonintegrated, transgene-free CB-derived hiPSC lines (CB-iPSC) that were generated at high efficiencies (∼1–4% of input cells) using a novel BMSC co-culture system [5] and a seven-factor EBNA-based episomal system (7F; *SOX2, OCT4, KLF4, MYC, NANOG, LIN28, and SV40 T antigen;* SOKMNLT) [1]. In designing this reprogramming system (**Fig.** **S1**), we capitalized on the principle that the innate epigenetic plasticity of hematopoietic progenitors [13]–[17] can be positively influenced by stem cell niche signals. We found that this optimized BMSC co-culture system provided soluble factors that preserved the short-term viability, as well as frequencies of CD34^+^CD45^+^ multipotent erythro-myeloid hematopoietic progenitors (*e.g.,* GEMM-CFU and BFU-e) following plasmid nucleofection, compared to continued culture with hematopoietic GFs alone (**Fig.** **1a** **, S2a–b**). However, this enhancement was due to increased survival, not increased proliferation since BMSC co-culture did not increase the percentage of CB progenitor cell proliferation compared to GF stimulation alone. The percentages of CB cells entering into S phase were comparable to short-term expansion with hematopoietic GFs alone, and also comparable to cultured adult fibroblasts (**Fig.** **1b**).

### Non-integrated Reprogramming to High Quality hiPSC was Strikingly more Rapid and Efficient in BMSC-primed CB Progenitors, and Unlike Fibroblasts or Keratinocytes Required Only Four Episomal Yamanaka Factors

To define conditions for optimized hematopoietic progenitor reprogramming, we tested the hypothesis that a stromal microenvironment that enhances hematopoietic self-renewal [39]–[41] would augment the episomal reprogramming efficiency of CB progenitors. We activated highly purified (>96%) CD34^+^CD45^+^ CB progenitors with hematopoietic GFs (on day -3) followed by plasmid nucleofection with episomes expressing defined factors three days later (on day 0; see reprogramming protocol schematic, **Fig.** **S1a**). Day 0 GF-activated CB populations contained few primitive CD34^+^CD38^−^ stem-progenitors, and consisted predominantly of lineage-committed (*i.e*., >95% CD34^+^CD38^+^, and >99% CD33^+^CD45^+^) progenitors on the day of nucleofection (**Fig.** **1c** **,** **S2a**). Day 0 CB was nucleofected with a single pulse of either four (4F; *SOX2, OCT4, KLF4, MYC*: ‘SOKM’) or seven (7F; SOKMNLT) episomal factors, and subsequently co-cultured with or without irradiated human mesenchymal BMSC for an additional 3 days. This was followed by plating of single +/− BMSC-primed CB cells on MEF (on day 3) for subsequent determination of reprogramming efficiency (**Fig.** **S3**). The episomal 4F and 7F reprogramming efficiencies of +/− BMSC-primed CD34^+^ CB progenitors were comparatively evaluated in parallel experiments against adult keratinocytes and fetal/adult fibroblasts 3–5 weeks later (**Fig. S1b**).

Rare, tightly-packed hESC-like colonies with sharply defined borders emerged from 7F episome-nucleofected keratinocytes and fetal/adult fibroblasts with slow kinetics (∼5–7 weeks following gene transfer), and with extremely low efficiencies, but comparable to previously reported non-integrated methodologies (*i.e*., <0.001% of input cells) [1]–[5]. Additionally, the majority of the rare episomal fibroblast-iPSC clones that did emerge was unstable, and often differentiated spontaneously following 1–2 subcloning passages. Pilot gene transfer experiments with GFP reporter constructs revealed that a poor CB nucleofection gene transfer efficiency (in the range of ∼10–20%) of our extremely large (∼15–18 kb) episomal constructs was a limiting factor (**Fig.** **S4**). Thus, poor episomal gene transfer likely partially accounted for the poor efficiencies of episomal reprogramming observed here and reported elsewhere [1], [3], [6].

Without BMSC priming, 7F and 4F-nucleofected CD34^+^CD45^+^ CB progenitors generated hESC-like colonies with significantly greater rapidity than fibroblasts and keratinocytes, and with efficiencies that were comparable to previously reported derivations of episomal 7F CB-iPSC (*i.e.,* ∼0.2–1.0% of input CB cells; **Fig.** **1d**) [4], [6]. In striking contrast, in greater than 10 independent experiments with pooled donor CB samples, BMSC priming reproducibly and significantly augmented the emergence of episomal CB-iPSC colonies (**Fig.** **1d**). Unlike fibroblasts, which did not yield episomal hiPSC with less than 7F [1]–[3], 4F reprogramming of BMSC-primed CB progenitors with a single pulse of a single episomal plasmid (pCEP4-EM2K) was even more robust than with equimolar DNA quantities of the 7F three-plasmid system. Type III [43] TRA^+^ AP^+^ hESC-like colonies emerged rapidly with 4F-nucleofected BMSC-primed CD34^+^ CB cells (as early as 7–14 days), and at significantly higher efficiencies than without BMSC co-culture (15–100x fold greater, with 5–20% reprogramming efficiencies per input CB cell; *p*<0.05).

Since our episomal gene transfer efficiency for day 0 CB cells was in the range of 10–20% (**Fig.** **S4**), these unprecedented reprogramming efficiencies in the same range (up to ∼20%) suggested the possibility that BMSC-priming may actually accelerate the conversion of the *majority* of successfully 4F-nucleofected BMSC-primed CB progenitors into CB-iPSC with an efficiency much higher than we and others previously reported [4]–[6]. Additionally, unlike episomal keratinocyte-iPSC, fibroblast-iPSC, and non-BMSC-primed CB-iPSC colonies, we noted that the majority (>90%) of BMSC-primed CB-iPSC clones maintained a stable undifferentiated hESC-like morphology with minimal spontaneous differentiation that permitted subsequent manual subcloning with minimal effort (**Fig.** **S5**). Finally, this efficient reprogramming system ultimately produced high quality non-integrated CB-iPSC lines with normal karyotypes, the ability to form tri-lineage cystic teratomas, and that possessed transcriptional signatures at low passages (p9–12) that were more akin to hESC than non-integrated episomal fibroblast- and keratinocyte-iPSC (**Figs. S6, S7, S8, S9, S10**).

### Mature Lineage-committed Myeloid Progenitors were More Efficient Targets of Episomal Reprogramming than Primitive Hematopoietic Stem-progenitors

**Figure 2:**
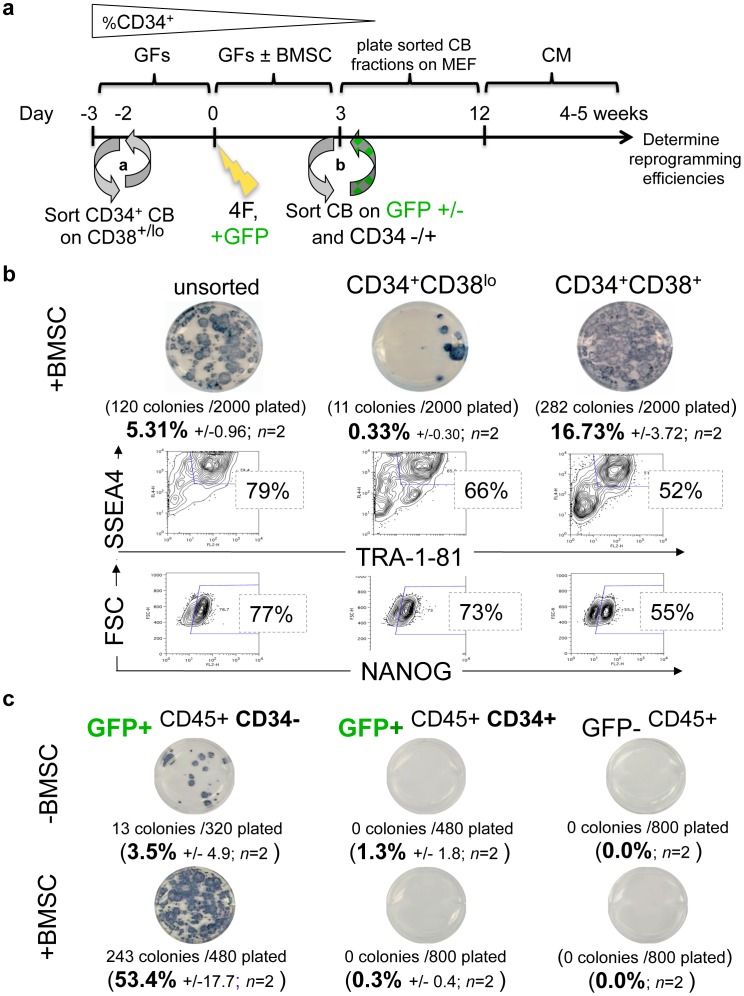
Episomal 4F reprogramming of FACS-purified hematopoietic populations. (**a**) Schematic summarizes the experimental strategy for determining 4F reprogramming efficiencies of FACS-purified hematopoietic progenitors of (**b**) BMSC-primed lineage-committed or (**c**) +/− BMSC-primed transgene-enriched myeloid populations. Experimental details are provided in Materials and Methods. The %CD34^+^ gradient symbol above the schematic reflects the concept that multipotent primitive CD34+CD38lo stem-progenitors are enriched during Days -3 to -2, but differentiate rapidly in culture thereafter (see also **Fig.** **1c** and **Fig.** **S2a)**. CD34^+^CD38^lo^ stem-progenitors give rise to lineage-committed CD34^+^CD38^+^ progenitors which subsequently differentiate further to CD34-negative CD33^+^CD45^+^ myeloid cells (*e.g*., promyelocytes). Post-sort analysis of FACS-purified Day 0 CD34^+^CD38^+^ CB fractions verified that >95% of this populations consisted of CD33^+^CD13^+^CD45^+^ myeloid cells (see also **Fig.** **1c**). Both reprogramming efficiency (AP and live TRA-1-81 staining of hESC-like colonies) and reprogramming completion (bulk SSEA4^+^TRA-1-81^+^ and NANOG^+^ FACS staining) assays were conducted 4 weeks following 4F nucleofections on P_0_ MEF cultures. (**b**) Shown is a representative AP staining (plates done in triplicate, with indicated average number of hESC-like colonies emerging per the number of single sorted day 3 BMSC-primed CB cells plated on MEF (i.e., unsorted CB *vs.* CD34^+^CD38^lo^ *vs.* CD34^+^CD38^+^ fractions. The averaged results of two independent experiments are indicated. In lower panels are shown representative FACS staining of surface TRA-1-81 and intracellular NANOG pluripotency markers of the same cultures demonstrating that 50–80% of AP^+^ hESC-like colonies possessed a Type III TRA-1-81^+^NANOG^+^ phenotype [43]. (**c**) To determine the more accurate reprogramming efficiency of purified +/− BMSC-primed episome-expressing myeloid populations, CB progenitors were co-nucleofected on day 0 with *both* the 4F pEP4 EO2S EM2K episome, as well as a pCEP4-GFP episomal GFP reporter construct. Episomal transgene expressing-only populations were subsequently FACS-purified by GFP expression prior to plating on day 3 MEF following with (+BMSC) or without (-BMSC) stromal co-culture. This was done by staining +/− BMSC-primed CB cells on day 3 with CD34-PE and FACS-purifying them into episome-expressing (GFP^+^CD34^+^, GFP^+^CD34^−^), and non episome-expressing (GFP^−^) populations prior to MEF plating. The results of AP stained plates shown are representative of independent sorting experiments using pooled donor CB samples for each 4F nucleofection, with the averaged results of two independent experiments indicated below. FACS analysis of these same experiments (data not shown) revealed 60% TRA-1-81 and NANOG expression for +BMSC AP^+^ colonies (GFP^+^CD34^−^), and 55% TRA-1-81 expression for –BMSC AP^+^ colonies (GFP^+^CD34^−^).

Previous studies suggested that stem-progenitors might possess an augmented propensity for pluripotency induction relative to differentiated somatic targets [6], [9]–[12]. To determine the nature of reprogramming of hematopoietic progenitors in our system, we tested the hypothesis that stem-progenitors within heterogeneous CD34^+^ CB populations were the target of efficient reprogramming. Thus, we FACS-purified CD34^+^ progenitors for episomal reprogramming at the initiation of our protocol (day -2.5) into stem-progenitor-enriched (CD34^+^CD38^lo^) or lineage-enriched (CD34^+^CD38^+^) fractions (i.e., prior to day 0 4F nucleofections and +/− BMSC priming (**Figs. 2a** **, S11b**). Post-sort FACS analysis verified that the purified Day -2.5 CD34^+^CD38^+^ lineage-enriched fraction consisted of >95% CD33^+^CD13^+^ myeloid progenitors (data not shown).

Unexpectedly, these experiments revealed that the Day -2.5 lineage-enriched (CD34^+^CD38^+^) fraction reprogrammed more rapidly, and generated significantly more AP^+^ colonies (16.73% +/−3.7 input efficiency; range: 13.0–20.4%, *n* = 2) than the more primitive Day -2.5 CD34^+^CD38^lo^ stem-progenitor fraction (0.33%+/−0.30 input efficiency) (**Fig.** **2b**). MEF wells that were seeded with as few as 2000 BMSC-activated CD34^+^CD38^+^-selected CB cells nucleofected with 4F routinely generated cultures containing 120–300 AP^+^TRA-1-81^+^ hESC-like colonies. Furthermore, bulk P_0_ cultures generated from these ∼300 to 2000 progenitors routinely produced >5×10^6^ reprogrammed cells that had already acquired Type III hiPSC expressions of NANOG and TRA-1-81/TRA-1-60 in 50–80% of all hESC-like AP^+^ colonies by 3–4 weeks following 4F episomal nucleofection (**Fig.** **2b**). Interestingly, although the more primitive Day -2.5 CD34+CD38^lo^-sorted population produced fewer AP^+^ colonies than the more committed Day -2.5 CD34+CD38+-sorted population per cell plated, the percentages by FACS of AP^+^ cells that were in completed NANOG^+^TRA^+^ reprogrammed states was qualitatively similar between the two groups (*i.e*., 50–70% in both groups).

### Purified CD34-negative Myeloid Cells Enriched for Expression of Episomal Transgenes Reprogrammed with an Optimized ∼50% Efficiency

A study that utilized transgenic mice expressing the Yamanaka factors homogenously in all somatic donor cells reported that hematopoietic stem and progenitor cells could be reprogrammed with efficiencies as high as 8–28% [11]. Since nucleofection efficiency was limiting in our episomal system (∼10–20% gene transfer efficiency; **Fig.** **S4**), we hypothesized that with further optimization, reprogramming in successfully-nucleofected cells may be even higher than the ∼15–20% efficiencies we observed in lineage-committed CB cells. To more accurately determine the reprogramming efficiency of transgene-expressing myeloid progenitors, we employed a strategy (see schematic; **Fig.** **2a**) that simultaneously enriched for both lineage-committed cells, *and* cells that had been successfully nucleofected with a single pulse of the large 4F episomal plasmid. CB cells were co-nucleofected on day 0 with a parental pCEP4-GFP construct along with the 4F episome (pEP4 EO2S EM2K). After three days of +/− BMSC co-culture, CD34-positive and CD34-negative episome-expressing progenitors were subsequently purified by FACS. Three Day 3 CB populations were sorted based on GFP and CD34 expression: GFP^−^, GFP^+^CD34^+^, and GFP^+^CD34^−^ expression (**Fig.** **2c**). These sorted Day 3 CB populations were then plated onto MEF for reprogramming efficiency determinations, as above. Unexpectedly, these experiments revealed that Day 3 CD34-negative (i.e., the CD33^+^CD45^+^-purified myeloid cells which arise directly from the CD34+CD38+ population) generated dramatically higher frequencies of AP^+^TRA-1-81^+^ hESC-like colonies compared to the less differentiated CD34-positive-purified fraction. Under these conditions, at least 50% of the episome-expressing (GFP+) CD34^−^CD45^+^ BMSC-primed lineage-committed CB cells rapidly converted to AP^+^ hESC-like colonies state. FACS analysis of these colonies further demonstrated that ∼60% of these colonies possessed a NANOG^+^TRA-1-81^+^ Type III phenotype (data not shown). Taken together, these data demonstrated for the first time, that homogenous expression of the four Yamanaka factors from a single pulse on one non-integrating plasmid was sufficient to reprogram the large majority of a sample of human myeloid cells, and without need for additional oncogenic factors (*e.g.,* SV40T Ag or LIN28^1^), repeated transfections, or mutagenic chromatin-modifying small molecules [44].

### GF-activated Myeloid Progenitors did not Endogenously Express Core Reprogramming Factors, but Instead Expressed High Levels of Common Epigenetic Regulatory Circuits

We next sought to identify the factors and mechanisms that mediated efficient pluripotency induction from myeloid progenitors. High endogenous expression of key Core factors (*e.g. SOX2*) was previously suggested to account for the relative ease of reprogramming observed in neural stem cells [9], [10]. However, quantitative real-time RT-PCR analysis of donor cell populations did not reveal increased endogenous expressions of known ESC-specific reprogramming factors (*e.g., SOX2, OCT4, LIN28, UTF1, NANOG, etc*) in day 0 hematopoietic progenitors from various sources (*e.g.,* FL, CB, mPB, BM, and CD34^+^CD38^+/lo^ -sorted CB) relative to fibroblasts and keratinocytes (**Fig.** **S11a**). While endogenous *MYC* and *KLF4* were expressed 6–30x-fold higher in day 0 hematopoietic progenitors compared to fibroblasts, such elevated expression levels were also observed in inefficiently reprogrammed keratinocytes. Thus, differences in expression of Core reprogramming factors did not appear to account *a priori* for the dramatic differences in reprogramming efficiencies observed between fibroblasts, keratinocytes, and CB progenitors.

**Figure 3:**
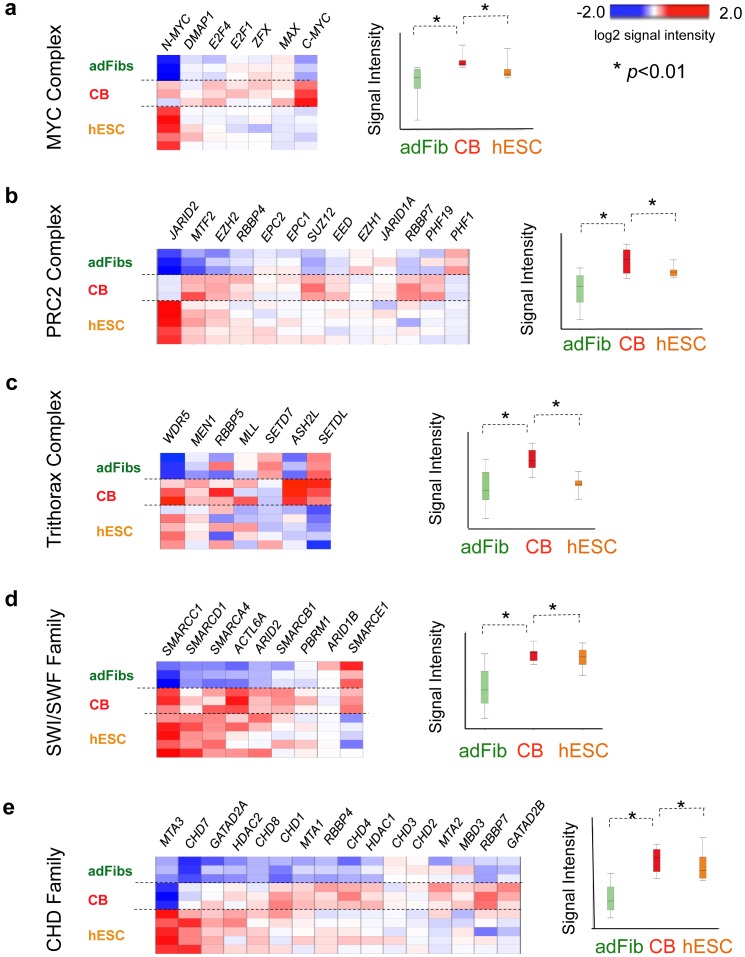
Endogenous expression of chromatin remodeling factors known to augment reprogramming efficiency in parental donor cells. Relative expressions of genes in chromatin remodeling factors in parental donor samples. (**a**) MYC complex regulators, (**b**) PRC2 complex regulators, (**c**) Trithorax complex regulators, (**d**) SWI/SWF family regulators, and (**e**) Chromodomain (CHD) regulators in day 0 GF-activated CB; pooled CB donors; *n* = 3 samples), adult fibs (adFibs; *n* = 3 samples), and hESC (*n* = 5 samples). Heatmaps and adjacent boxplots show log2 mean-subtracted normalized values of signal intensities from averaged, independent biological replicate microarray samples (n = 3–5 per condition) averages of values, with *p<0.05 where indicated. In mean normalization, each gene’s mean log2 signal value is determined for all the cell types, and then subtracted from each cell type’s signal intensity value for that gene.

To gain further insight, we shifted our focus from single reprogramming factors to wider transcriptional circuits that have been validated to direct efficient pluripotency induction. We evaluated the expression of pluripotency-mediating networks at sequential stages of our CB reprogramming protocol via microarray analysis and a previously described modular bioinformatics approach [26], [27]. These studies revealed that relative to adult fibroblasts and unstimulated day -3 CB, day 0 GF-activated CB progenitors robustly expressed a broad palette of chromatin remodeling factors that are known to experimentally enhance iPSC generation (*e.g.,* members of the MYC [27], Polycomb (PRC2, PRC1) [31]–[34], Chromodomain (CHD) [36], SWI/SWF [37], and Trithorax [38] complex families) (**Fig.** **3**). These transcription factor complexes are expressed in both ESC and hematopoietic stem-progenitors and are at the upper tier of networks that regulate epigenetic plasticity, self-renewal, and lineage specification in *both* cell types. The downstream networks these factors control include the MYC complex-regulated transcriptional circuits (*e.g.,* the ‘ESC module’ [26], and the recently described MYC module [27]), as well as bivalent Polycomb group (PcG)-regulated circuits (*i.e.,* PRC1 [34], PRC2 [32]–[34] modules) which repress lineage specification (**Table S1**). Interestingly, these regulatory factors were expressed in GF-activated day 0 CB cells at mean levels that were even higher than hESC (**Fig.** **3**). These data suggested an alternative etiology for the efficient reprogramming of CB myeloid progenitors: that conversion to pluripotency was facilitated not by endogenous somatic expression of key Core reprogramming factors, but instead by a molecular infrastructure of poised pluripotency-associated regulatory circuits (*e.g.,* ESC, MYC, PRC1, PRC2 modules) that become activated by hematopoietic GFs (*e.g.,* Flt3L and TPO).

### ESC-like MYC and Polycomb (PcG)-regulated Circuits were Expressed *de novo* in Partially Reprogrammed States that were Rapidly Reconfigured from Hematopoietic to hESC-like Patterns Following Episomal Factor Expression

**Figure 4:**
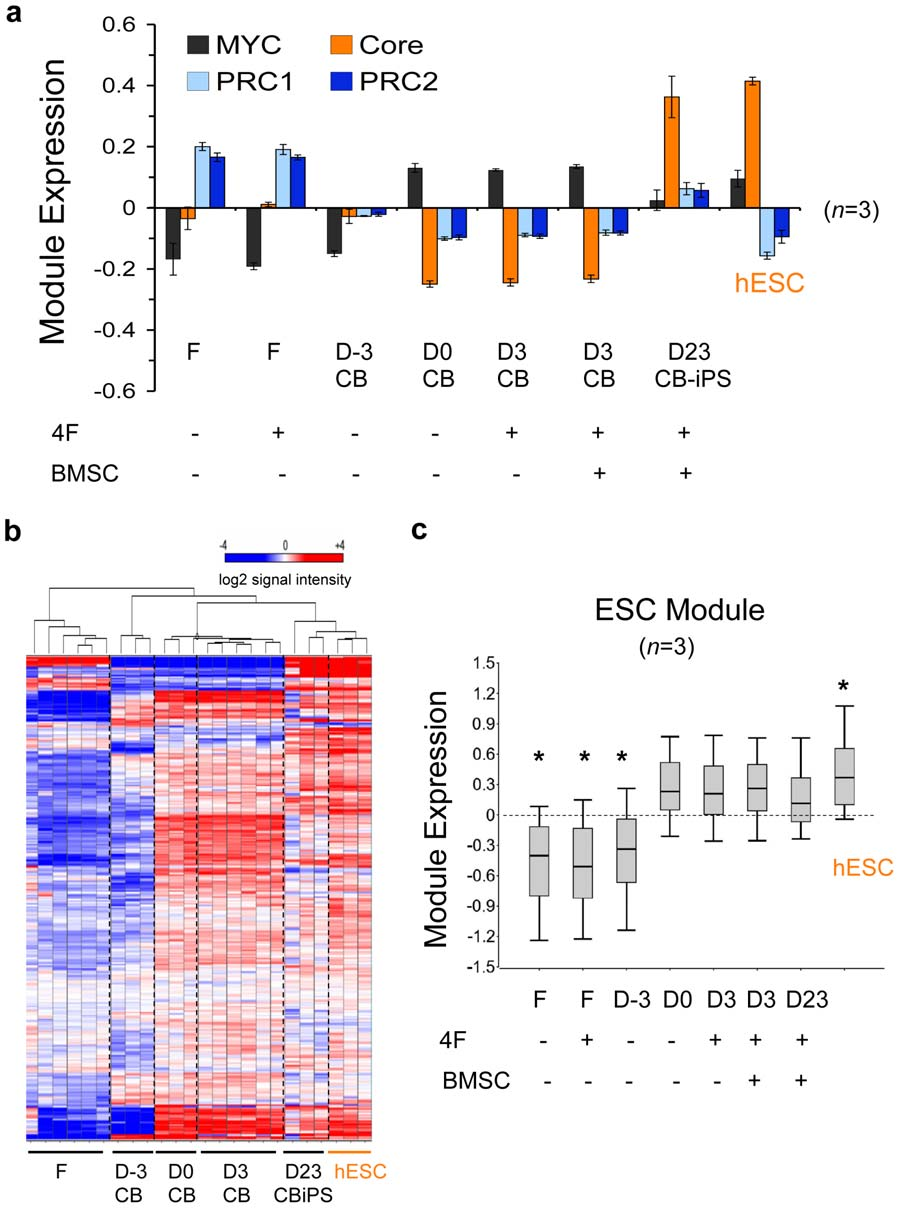
Partially reprogrammed stem cell modules in GF-activated CB myeloid progenitors are rapidly reconfigured to hESC-like patterns. (**a**) Illumina microarray expressions of ESC-like gene modules (MYC, PRC1, PRC2, and Core; see **Table S1** for gene lists). Shown are donor cell populations with (+) and without (−) 4F episomal nucleofections, and with (+) and without (−) BMSC-priming: adult fibroblasts (F), Day -3 unstimulated CB cells (D-3 CB), Day 0 FTK GF-stimulated CB cells (D0), Day 3+/− BMSC-primed (D3), and bulk Day 23 early CB-iPSC culture (D23 iPSC). These early D23 iPSC populations were already composed of >50–60% populations with fully-reprogrammed TRA-1-81^+^NANOG^+^ phenotypes. Undifferentiated H9 hESC samples served as control (hESC). Module expressions represent log2 mean-subtracted normalized values of signal intensities from averaged, independent biological replicate microarray samples (n = 3 per condition). Although they possessed a transcriptionally inactive Core module, day 0 GF-activated CB progenitors expressed active ESC and MYC modules, and inactive PRC1, and PRC2 modules at mean expression levels that were already comparable to levels in hESC. The annotation and references of all genes in each module is provided in **Table S1**. (**b**) Partially reprogrammed ESC module [26] expression in CB progenitors. Legend for each sample is the same as above. Unsupervised hierarchical clustering heatmaps of (**b**) expression and (**c**) Box plots of log2 mean-normalized values of the ESC module gene signal intensities in somatic target populations, hESC, and reprogrammed cell lines. The heatmap’s color scale was chosen to emphasize subtle mid-range change. The resulting values emphasize relative expression across cell types rather than relative absolute expression across genes. This box and whisker plots (right panels) depict the log2 mean-subtracted normalized values of signal intensities of genes comprising the module set for each cell type indicated from Illumina array data. The top and bottom of a box mark the 75^th^ and 25^th^ percentile log2 signal values, respectively, while the bar at the middle denotes the median. The whiskers above and below each box mark the upper 90^th^ and lower 10^th^ percentiles. Paired tests with significance *p*<0.05 (*) or without significance (*NS*; p>0.05) with values of control hESC are indicated.

We next sought to correlate the modular expressions of ESC-like networks to the observed reprogramming efficiencies of CB progenitors and fibroblasts. Module expressions were quantified before and after episomal expression in donor fibroblasts and CB at sequential phases of reprogramming: day -3 naïve unprimed CB (D-3), day 0 GF-primed CB (D0), day 3+/− BMSC-primed CB samples (D3), and newly emerged Day 23 bulk CB-iPSC cultures, which consisted of majority (50–80%) populations of NANOG^+^TRA^+^ cells (**Fig.** **2a**). These analyses revealed that relative to fibroblasts and day -3 GF-unstimulated CD34^+^ CB cells, day 0 GF-activated CB progenitors expressed strongly *activated* ESC-like MYC-regulated modules (MYC, ESC), and *inactivated* ESC-like PcG complex-regulated modules (PRC1, PRC2) (**Fig.** **4a**). Although all samples including CB possessed a transcriptionally *inactive* Core module, the mean expression levels of day 0 GF-activated CB progenitor ESC, MYC, PRC1, and PRC2 modules were already comparable to levels in hESC. In contrast, fibroblasts did not possess hESC-like levels of expression for any of these pluripotency-regulating circuits. These data revealed that activation by GFs alone was sufficient to induce a cascade of ESC-like circuits in CB progenitors. We also noted that the composite modular expression pattern of activated CB progenitors was identical to the ‘partially reprogrammed’ iPSC state previously described by Orkin’s group [27]. This transcriptional state consists of activated ESC-like expression levels of MYC- regulated and inactivated PcG complex-regulated modules, and requires only activation of the Core module to complete somatic induction to a stable pluripotent state.

Collectively, these modular bioinformatics studies revealed several important principles regarding CB reprogramming: 1) CB cells begin with hESC-like quiescent PcG-regulated module expression, 2) GF stimulation subsequently activates MYC-regulated modules (ESC, MYC) to hESC-like levels without affecting Core module expression, 3) GF-activated day 0 CB MYC- and PcG-regulated circuits rapidly reconfigured from hematopoietic to hESC-like transcriptional patterns by day 23 of the reprogramming system, with concomitant activation of the SON-regulated Core module (i.e., following Core factor expression from the 4F episome; **Fig.** **4b,c**). These data also revealed that the mechanism by which BMSC priming further augments reprogramming efficiency was likely independent of initial MYC-regulated module activation, since BMSC priming did not further enhance the conversion of Day 0 gene module activities at Day 3.

### Magnitude of Expression of MYC-regulated and OCT4-associated Modules Correlated with and Directly Predicted High Reprogramming Efficiencies

**Figure 5:**
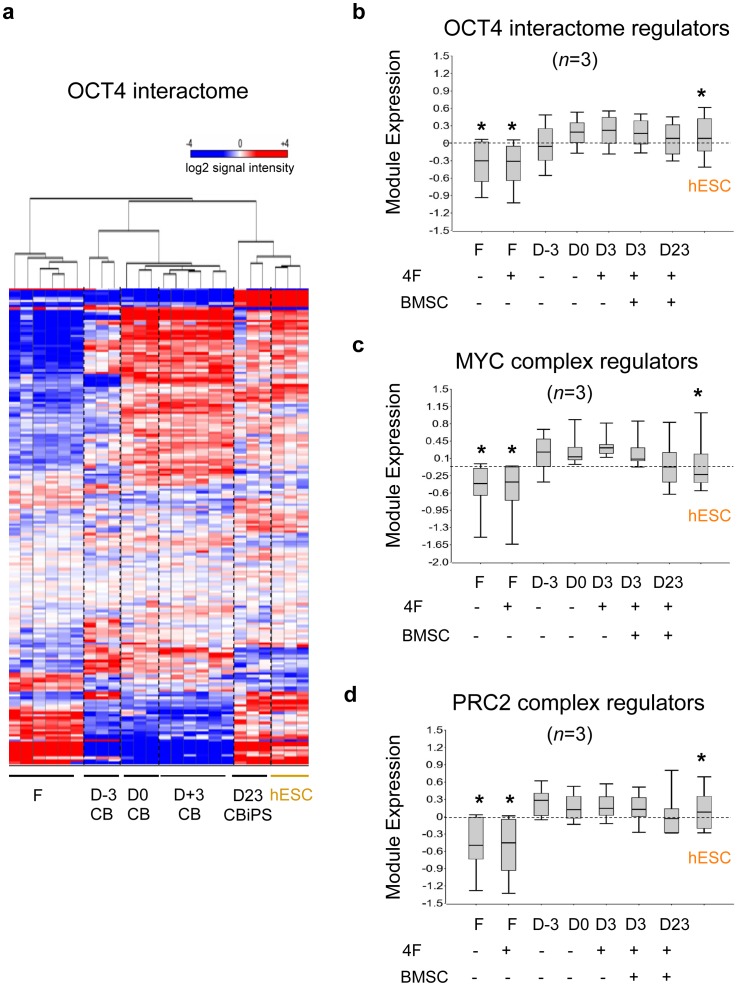
GF-activated progenitors expressed an active OCT4-associated network as well as chromatin remodeling factors known to augment iPSC generation. (**a**) Unsupervised hierarchical clustering heatmap of expression of the OCT4 interactome module [24] (**Table S1**) in fibroblasts (F), CB progenitors at various stages of the reprogramming protocol (D-3, D0, D3+/− BMSC), day 23 bulk early CB-iPSC cultures (D23 iPSC), and hESC (H9) controls. Gene arrays samples (*n* = 3 per condition) are the same as defined above. Box and whisker plots of same samples of the log2 mean-subtracted normalized values of signal intensities of gene module sets for (**b**) a subset of the OCT4 interactome consisting of ESC-regulating epigenetic modulators [24] (see **Table S1** for list), (**c**) the MYC transcription factor complex [27] (*N-MYC, C-MYC, E2F4, E2F1, ZFX, MAX*), and (**d**) the PRC2 repressive complex [32] (*JARID2, MTF2, EZH2, RBBP4, EPC2, EPC1, SUZ12, EED, EZH1, JARID1A, RBBP7, PHF19, PHF1*).

Our observation that GF-activated hematopoietic progenitors already expressed multiple active ESC-like circuits and epigenetic remodeling factors posed the possibility that a wider and more organized pluripotency-associated framework existed in GF-activated myeloid cells. For example, the critical Core reprogramming factor OCT4 physically interacts not only with its Core factor partners (e.g. SOX2 and NANOG), but also with a known, defined supportive protein network (the ‘OCT4 interactome’) [21]–[24] that regulates transcription, DNA repair, DNA metabolism, and chromatin modification (*e.g.,* PRC1, SWI/SWF, NuRD, CHD, Trithorax complexes). Using a modular approach, as above, we measured the transcriptional activity of this OCT4-associated circuit, as well as several other epigenetic regulator families that experimentally enhance iPSC generation and maintain the pluripotent state (e.g., MYC and PRC2 complex regulators; see **Table S1**). Strikingly, in contrast to fibroblasts and unstimulated day -3 CB cells, day 0 GF-activated CB progenitors robustly over-expressed this OCT4-associated network (**Fig.** **5a**) including its epigenetic regulator component (**Fig.** **5b**), as well as the MYC complex (**Figs. 3a** **,** **5c**) and PRC2 regulator repressor complex (**Figs. 3b** **,** **5d**), which have all been experimentally validated to be indispensable for pluripotency as well as facilitating somatic reprogramming. These data further validated our working hypothesis that ESC-like networks, including activated OCT4-interacting circuits likely regulate similar processes of self-renewal and lineage specification in both hematopoietic progenitors and ESC [42].

**Figure 6:**
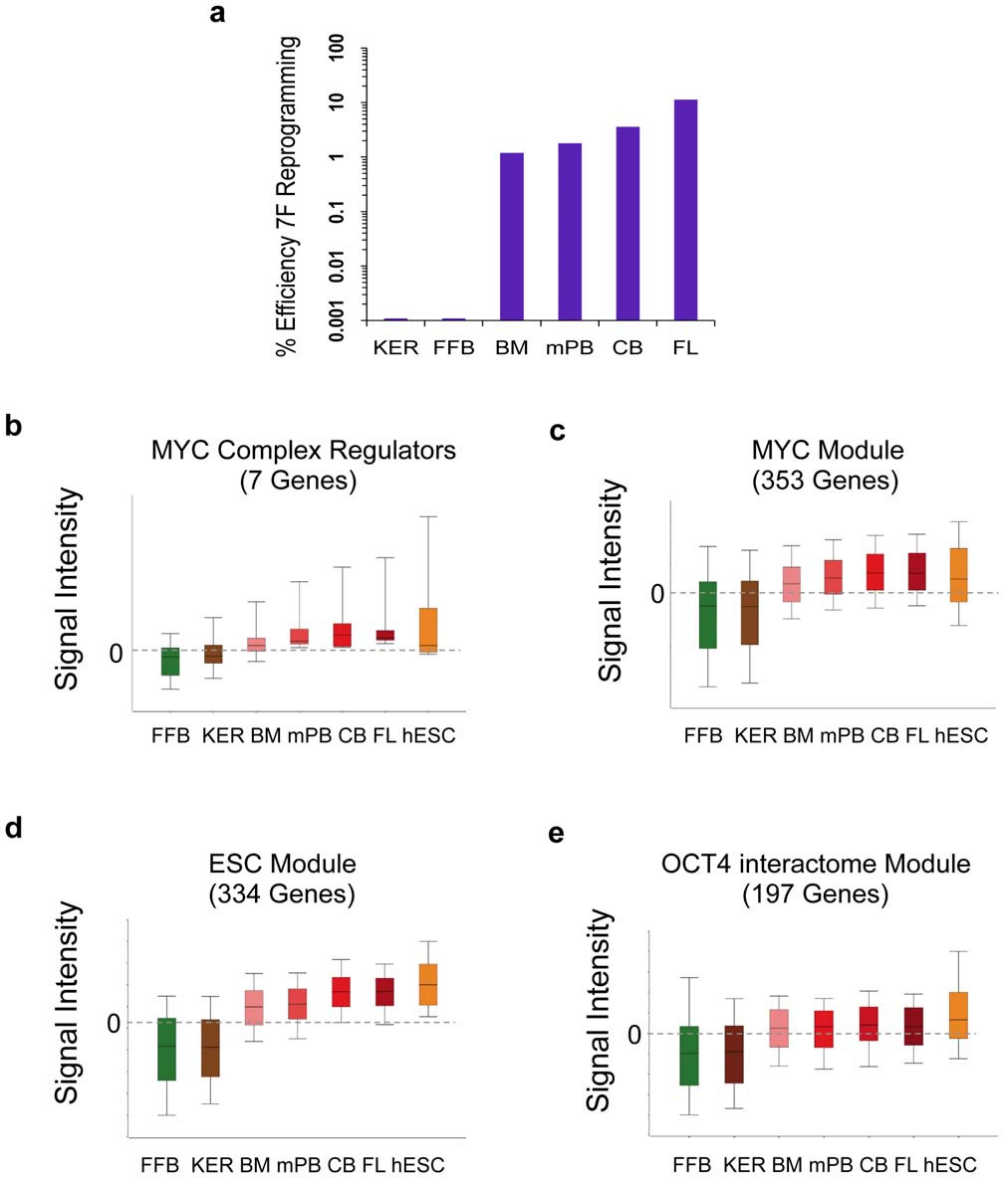
Reprogramming efficiency in developmentally progressed GF-activated hematopoietic progenitors is predicted by and directly correlates with expression levels of ESC-like circuits. (**a**) 7F (SOKMNLT) reprogramming efficiencies of developmentally progressing BMSC-primed GF-activated Day 0 progenitors were determined with AP^+^ staining staining of ESC-like colonies, at P_1_, 25 days following 7F nucleofections. BMSC-primed Day 0 CD34+ hematopoietic progenitors (FL, CB, mPB, BM), keratinocytes (KER), or fetal fibroblasts (FFB) populations were reprogrammed with non-integrated 7F episomes as described in text. Log2 mean-normalized microarray expressions (signal intensities) in somatic target populations (from pooled Day 0 GF-primed CD34^+^ donors; n = 3–4 per sample) and H9 hESC of (**b**) MYC complex regulator genes, (**c**) MYC module, (**d**) MYC-regulated ESC module, and (**e**) OCT4 interactome gene module (see **Table S1** for gene module members).

To determine if the magnitude of expression of these circuits directly correlated with and could predict the magnitude of reprogramming efficiency in our somatic donor cells, we computed the expression levels of ESC-like modules in our donor cells. GF-activated day 0 progenitors from progressive stages of CD34^+^ developmental maturity (*i.e.*, 20–22 week-old fetal liver (FL), neonatal CB, adult GCSF-mobilized peripheral blood (mPB), or adult bone marrow (BM) as well as fibroblasts and keratinocytes were all assayed for their comparative reprogramming efficiencies with 7F episomes (**Fig.** **6a**). Regardless of hematopoietic source, hESC-like colonies with high AP staining and surface TRA-1-81 expression were generated at significantly higher efficiencies compared to episomally-nucleofected keratinocytes and fibroblasts. The efficiency of hESC-like colony generation correlated exactly with the developmental stage of the hematopoietic progenitor, with a hierarchy of reprogramming rate and efficiency: FL> CB > adult mPB> adult BM. A computation of mean expressions across all of our somatic targets of the MYC regulator complex [27] and its downstream gene module targets (ESC, MYC modules), as well as the OCT4 interactome gene module revealed a pattern that correlated identically with and predicted the observed hierarchy of hiPSC reprogramming efficiencies of somatic targets: FL> CB > adult mPB, adult BM > keratinocytes, fibroblasts (**Fig.** **6b–e**).

### Factor-driven Conversion to a Pluripotent State was Further Accelerated by Soluble and Contact-dependent Stromal Signals

**Figure 7:**
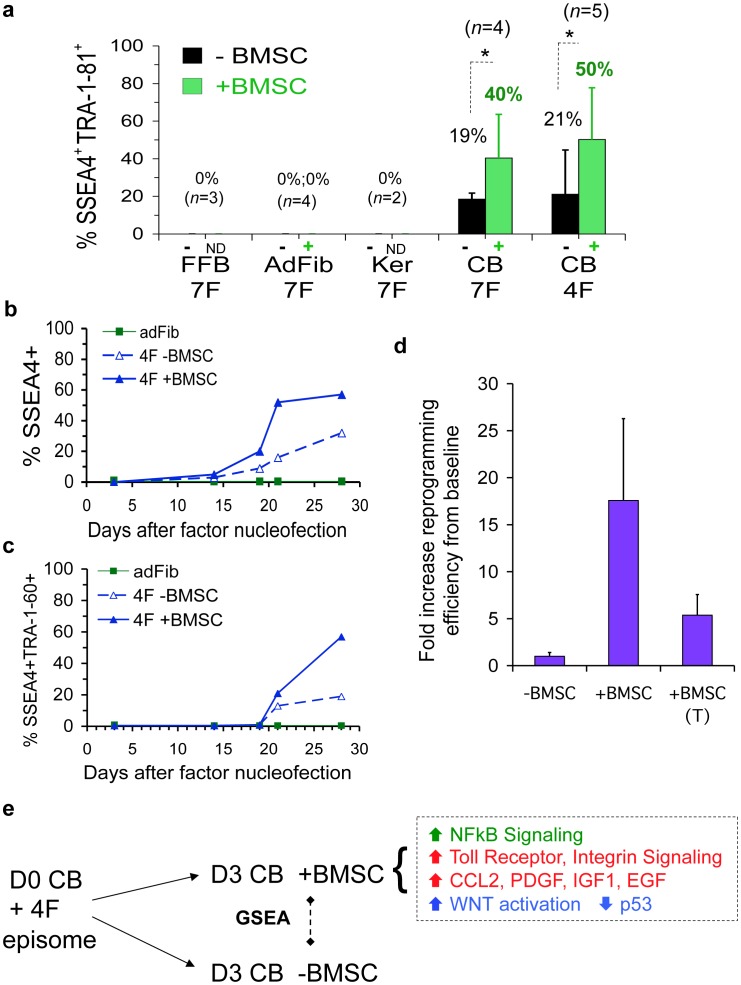
Episomal reprogramming was further accelerated by paracrine and contact-dependent stromal signals. (**a**) Emergence of surface pluripotency markers (SSEA4, TRA-1-81) were assayed by FACS at 3 weeks in bulk cultures of 4F episomally-reprogrammed somatic cells briefly co-cultured with (+) or without (**−**) irradiated BMSC (**Fig.** **S1**). Fetal fibroblasts (FFB), adult fibroblasts (AdFib), adult keratinocytes (Ker), and GF-activated Day 0 CB (CB) were nucleofected with 4F or 7F on Day 0 (**Fig.** **S1**), and reprogrammed bulk cultures were analyzed by FACS 3 weeks later. AP stains of hESC-like colonies were done in parallel of these same experiments, and are presented in **Fig.** **1d**. Shown are the averaged results of 2–5 experiments with averages, and significances (*) designated at peak of bar graphs. (**b,c**) The kinetics of pluripotency marker emergence of 4F reprogrammed CB progenitors with (+) and without (−) BMSC priming. (**b**) SSEA4^+^, and (**c**) SSEA4^+^TRA-1-60^+^ expressions. (**d**) Enhancement of 4F CB reprogramming with (+BMSC) and without (- BMSC) stromal priming was due to signals that were partially cell contact-dependent, and partially soluble factor-mediated. GF-activated CB cells were cultured as described in **Fig.** **S1** from Day 0 until Day 3 without BMSC co-culture (-BMSC), with BMSC co-culture (+BMSC), or with BMSC co-culture but physically separated from CB cells with a Transwell insert that prevented cell-cell contact between BMSC and CB cells, but allowed diffusion of soluble stromal factors (+BMSC (T)). Shown is the relative fold-increase of reprogramming efficiency (enumerated AP+ hESC-like colonies) from two averaged 4F-reprogramming experiments from baseline efficiencies (-BMSC conditions). Reprogramming efficiency was determined at 3 weeks post-nucleofection with 4F, determined by AP staining of hESC-like colonies (as described in Methods). (**e**) Gene specific enrichment analysis (GSEA) computation of pathways activated in 4F-nucleofected CB cells by stromal signals. The GSEA algorithm was used to identify curated pathways over-represented among genes with significant (p<0.05, FDR<0.05) differential expression between Day 3 (D3) +BMSC-primed CB samples, *vs*. D3 unprimed (-BMSC) CB samples that were nucleofected at Day 0 with 4F episome. **Table S3** summarizes the MSigDB v. 3.0 gene set categories that were enriched with FDR <0.05 and nom *p*<0.05 for these two paired gene set computations. Shown in (**e**) are the major categories of enriched pathways that were significantly and differentially activated in Day 3 BMSC-primed and 4F-nucleofected CB samples.

Our modular bioinformatics approach gave insight into how GF activation of ESC-like modules in myeloid cells may drive a more efficient reprogramming than fibroblasts or keratinocytes. However, it did not enlighten how BMSC priming further augmented this baseline efficient pluripotency induction by an additional 20–100–fold, since the expressions of ESC-like modules were not further enhanced by stromal priming, at least by day 3 of our reprogramming system (**Figs. 4** **,** **5**). Thus, we next investigated the general mechanism by which stromal signals might further augment the reprogramming of myeloid progenitors with pre-activated ESC-like circuits. Bulk FACS analysis at three weeks of the AP^+^ hESC-like colonies that developed from our various somatic donors revealed that BMSC priming significantly (*p*<0.05) enhanced the attainment of a phenotypically fully reprogrammed state in the great majority of early 4F and 7F CB-iPSC (*i.e.,* 50–80% of cells expressed SSEA4^+^TRA-1-81^+^) (**Fig.** **7a**). A kinetic analysis of the emergence of pluripotency marker SSEA4 and TRA expression with and without BMSC co-culture during the first 4 weeks of 4F CB reprogramming revealed that brief stromal co-culture indeed *accelerated* the kinetics of emergence of pluripotency marker-positive populations following 4F expression (**Fig.** **7b,c**). To probe the relative roles played by cell extrinsic paracrine *vs.* contact-dependent stromal signals, CB reprogramming experiments were performed with tissue culture Transwell inserts that physically separated stromal layers from nucleofected CB cells (but allowed transfer of diffusible stromal-derived factors). These Transwell studies revealed that CB reprogramming by BMSC was enhanced by complex stromal signals that were partially contact-dependent *and* partially soluble factor-mediated (**Fig.** **7d**).

To identify putative soluble factors generated by CB-BMSC interactions that might be augmenting reprogramming efficiency, supernatants from day 3 CB cultures in our reprogramming system co-cultured with or without BMSCs were harvested and subjected to antibody array proteomic analysis (**Table S2**). These studies detected a differentially- enriched presence in the CB-BMSC secretome of multiple stem cell growth factors known to support both HSC and ESC self-renewal. These included chemokines and growth factors that signal through JAK-STAT3 (*e.g.,* PDGF, IL-6, GCSF, CCL2), as well as WNT ligands, BMPs, and FGFs. Interestingly, a large fraction of the soluble factors differentially enriched in CB-BMSC supernatants were those known to be associated with activation of Toll receptor or NFκB signaling.

To gain broader insight into unique molecular pathways that might be differentially activated in myeloid progenitors by the stromal microenvironment, we evaluated the differential genome-wide expression patterns of purified 4F-nucleofected day 3 CB cells co-cultured with and without BMSC priming using Gene Set Enrichment Analysis (GSEA) computation (**Fig.** **7e**, **Table S3**) [45]. This analysis of the microarray data revealed a highly significant (FDR<0.05) differential activation of multiple key contact-dependent and growth factor-mediated pathways in day 3, BMSC-primed, 4F-nucleofected CB cells. These data included both unexpected as well as previously implicated pathways that are known to potentiate both hematopoietic self-renewal *and* ESC pluripotency, as well as facilitate induced pluripotency. The pathways that were enriched with high statistical significance (*p*<0.001, FDR<0.01) in BMSC-primed CB myeloid cells included: 1) pathways that activate WNT signaling [46], destabilize p53 [7], or regulate cell cycle transition, 2) soluble and contact-dependent pathways that signal through JAK-STAT3 (*e.g.,* PDGF, integrins [47]), and unexpectedly (yet consistent with our CB-BMSC supernatant proteome studies): 3) chemokine and anti-apoptotic cascades activated by Toll receptor-NFκB signaling [48], [49].

## Discussion

**Figure 8:**
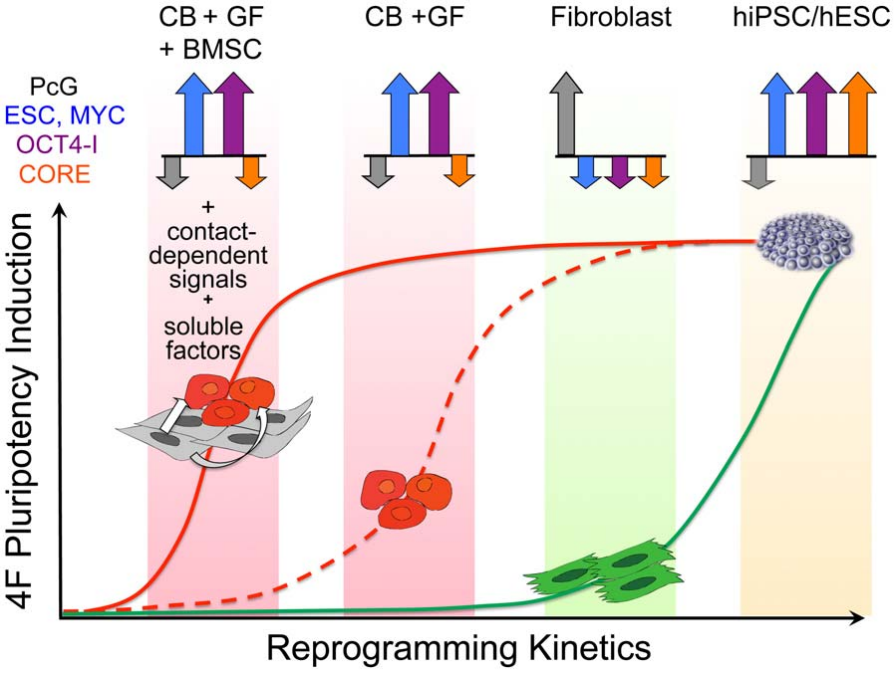
Efficient episomal reprogramming of human myeloid progenitors is mediated by synergies between extrinsic stromal signals and the GF activation of partially reprogrammed ESC-like gene modules. MYC; MYC-regulated ESC and MYC gene modules, OCT4-I; OCT4 interactome gene module, PcG-regulated targets; PRC1, PRC2 gene modules, Core; SOX2-OCT4-NANOG-regulated gene module (see **Table S1** for gene lists that comprise each module). Day 0 GF-activated CB progenitors expressed *de novo* activated MYC and OCT4-I (but not Core) gene modules and inactivated PcG-regulated modules (PRC1, PRC2) at hESC-like levels. These networks were subsequently facilitated to a stable pluripotent state (and a completion of reprogramming of the Core module) via synergies between stromal signals and transient episomal expression of the Yamanaka factors.

We have developed and characterized an optimized reprogramming system that generates high quality hiPSC from human myeloid progenitors with unprecedented efficiency. To our knowledge, this study is the first to identify important synergies between hematopoietic regulatory circuits activated by GFs and extrinsic niche factors to efficiently direct the pluripotency induction of lineage-committed myeloid cells. Efficient reprogramming correlated not simply to endogenous expression of individual Core reprogramming factors (*e.g.,* SOX2, OCT4, NANOG), or their downstream Core module targets, but to ESC-like expression levels of epigenetic regulators (*e.g.,* PcG/PRC2 complex, MYC complex, and Trithorax complex) and their downstream targets (*e.g.,* ESC, MYC, PRC1, and PRC2 modules). Collectively, these ESC circuits were poised in partially reprogrammed expression states following GF activation of myeloid progenitors, prior to ectopic episomal Yamanaka factor expression (**Fig.** **8**). The episomal reprogramming efficiency of BMSC-primed progenitors, which was ∼4–10% in unfractionated day 0 CB cells, and ∼50–65% in purified episome-expressing myeloid cells was 4 to 5 logs greater than that of fibroblasts or keratinocytes. Our GFP purification experiments demonstrated that AP^+^ hESC-like colonies were emerging not from a minority population, but from the majority of episome-expressing myeloid cells. Moreover, the great majority (∼50–80%) of these hESC-like colonies had already achieved a fully reprogrammed NANOG^+^TRA-1-81^+^ (Type III hiPSC) phenotype by 3–5 weeks following 4F or 7F episomal nucleofection. The application of methods with higher gene transfer efficiencies for expressing reprogramming factors (*e.g*. via synthetic mRNAs or microRNAs) may allow further optimization of this hematopoietic reprogramming system. More importantly, this experimental system opens new avenues of molecular, epigenetic and proteomic investigations for elucidating novel micro-environmental factors that drive rapid and efficient reprogramming in synchronized populations of donor cells in more defined conditions.

Our demonstration that lineage-committed CD33^+^CD45^+^CD34^−^ myeloid cells, and not immature hematopoietic stem-progenitors were more efficiently reprogrammed contrasts and refines the conclusions of previous studies that suggested primitive stem-progenitors are the most amenable cell type for factor-driven reprogramming [6], [9]–[12]. In an inducible transgenic ‘secondary system’, Eminli et al [11] similarly observed that lineage-committed myeloid progenitors (*e.g*., GMP, CMP, and MEP populations) had the highest reprogramming potential of all hematopoietic cell types (including primitive HSC), and that this propensity was not directly related to their proliferative status. In agreement with our findings, several somatic cell nuclear transfer reprogramming studies also reported that undifferentiated HSC did not possess a greater cloning efficiency than differentiated myeloid cells [51], [52]. Although, the cellular and molecular nature of facile myeloid reprogramming requires further investigation, it is likely related to the unique epigenetic plasticity of hematopoietic progenitors which consists of a CpG methylome, a highly dynamic chromatin structure (*e.g.* transcriptionally-active (H3-K4me3), and repressive (H3-K27me3) histone marks [14]–[17]) that may be similar in configuration to ESC. Collectively, our results are consistent with the notion that the genome and epigenome of committed myeloid progenitors possess a transcriptionally permissive ESC-like state. The Yamanaka factors appeared to cooperate with soluble and contact-dependent stromal signals to further accelerate conversion of GF-activated ESC-like myeloid circuits to a stable pluripotent state. Accordingly, bone marrow stroma are known to increase the ‘stemness’ of *in vitro* cultured hematopoietic progenitors by increasing expression levels of enzymes and pathways involved in self-renewal (*e.g., DNMT3A, DNMT3B, TERT,* NOTCH, and WNT), and by promoting epigenetic ESC-like histone modifications [39]–[41]. Our systematic GSEA of BMSC-primed myeloid cells confirmed that stromal priming differentially activated multiple soluble and cell contact-dependent pathways that have previously been previously implicated in potentiating ESC self-renewal and induced pluripotency including WNT activation, p53 destabilization, and integrin signaling [46]–[48]. Additionally, BMSC-primed CB cells possessed unexpected, yet statistically significant molecular activation of Toll receptor and NFκB signaling pathways, which have previously been implicated in regulating pluripotency [49]. However, the mechanism by which activation of NFκB signaling by BMSC priming mediated augmentation of factor-driven reprogramming in myeloid cells remains to be elucidated. Interestingly, a recent study has independently discovered a role for TLR signaling in driving efficient viral vector-mediated reprogramming [50].

An important theme unveiled from our studies was that efficient induced pluripotency might require extrinsic micro-environmental activation of a molecular framework that commonly regulates self-renewal and differentiation in both hematopoietic progenitors and ESC. Hematopoietic progenitors and ESCs share common transcriptional programs that allow them to self-renew while remaining poised to differentiate into multiple cell lineages [16], [31]–[34]. In ESC, self-renewal is regulated by concerted networks that include active MYC-regulated circuits, inactive Polycomb-regulated circuits, and an OCT4-interacting epigenetic network [24]. The generation of iPSC by defined factors requires the epigenetic activation of all of these transcriptional networks [27], which are all normally quiescent in somatic fibroblasts. We demonstrated that GF-activated myeloid progenitors already possessed high hESC-like expression levels of all these regulatory circuits (except the Core module) in partially reprogrammed transcriptional states (**Fig.** **8**). These pre-activated networks were rapidly reconfigured from hematopoietic to ESC-like transcriptional patterns following episomal expression of the Yamanaka core factors (*e.g.* OCT4, SOX2). Furthermore the reconfiguration of these *de novo* activated circuits networks to hESC-like patterns was dramatically accelerated by extrinsic stromal signals with an unprecedented bulk efficiency and rapidity that was limited only by the efficiency of episomal gene transfer.

MYC and its downstream transcriptional networks (ESC and MYC modules) appeared instrumental for efficient myeloid reprogramming, consistent with its role as a master regulator of chromatin modification, and its maintenance of the pluripotent state through effects on histone acetyltransferases [25]–[27]. Additionally, activation of MYC and its networks is a known downstream target of JAK-STAT3 signaling by the GFs implicated in these studies (*e.g*., FLT3, TPO, PDGF, CCL2). Furthermore, many of the 334 genes in the MYC-regulated ESC module are established transcriptional regulators of HSC self-renewal (*e.g*., MYC, DNMT1, and HDAC1). However, the promoters of many of these ESC module genes also bind the ESC-specific Core factors (SOX2, OCT4, and NANOG; SON ), thus likely providing a link for cross-regulation of Core and PcG networks [28]–[30]. Moreover, MYC-regulated circuits were progressively expressed in hematopoietic progenitors of decreasing maturity, thus providing one basis for the known augmented reprogramming efficiency seen in developmentally immature somatic cells.

Unexpectedly, GF-activated myeloid cells also expressed ESC-like levels of an organized OCT4-associated circuit [22]–[24] that is known to interact directly and support the function of the Core reprogramming factors in ESC. The role and function of this OCT4-interacting regulatory circuit has not yet been described in any class of stem cell or progenitor other than ESC. In a variety of somatic cells including fibroblasts, the activation of the Core network by defined factors is a rate-limiting step, and requires the additional epigenetic activation of a large auxiliary network of DNA and chromatin modifying proteins. These networks physically interact with OCT4 and NANOG in a protein-protein interactome to support pluripotency [22]–[24]. This OCT4 interactome includes a wide repertoire of ESC factors that have been experimentally validated to facilitate iPSC generation (*e.g.,* PcG, SWI/SWF, Trithorax, NURD, Chromodomain, LSD1 complexes), and that interact directly with the Core factors to regulate self-renewal, lineage fate, and promotion of hyper-transcriptional chromatin. The surprising observation that an OCT4-associated network was already active in GF-primed CB myeloid progenitors suggests a common regulatory biology of self-renewal and differentiation in both ESC and hematopoietic progenitors. Consistent with our hypothesis that myeloid progenitors already possess the required molecular infrastructure of induced pluripotency, but lack only activation of core circuits (by ectopic episomal core factor expression), previous studies demonstrated that CB progenitors can be reprogrammed (albeit inefficiently) with only SOX2 and OCT4 transgenes [12].

In summary, this highly optimized human myeloid reprogramming system provides a new paradigm for understanding the augmented reprogramming capacity of somatic progenitors. Detailed elucidation of the stromal signals that augmented myeloid reprogramming should facilitate highly efficient reprogramming in defined conditions for other cell types, thus obviating the need for stromal priming. Further dissection of this experimental system may also unveil a common biology that regulates hematopoietic progenitors and pluripotent stem cells.

## Materials and Methods

### Ethics Statement (Human Embryonic Stem Cell Lines)

The hESC lines used in this proposal served as controls for hiPSC experiments, and were originally obtained commercially from the WiCell Research Institute (Wisconsin International Stem Cell Bank; WISCB). The use of all WISCB-donated hESC lines in these studies was approved by the Johns Hopkins Embryonic Stem Cell Research Oversight (ESCRO) Committee, and conform strictly to Johns Hopkins University Institutional standards, including written informed consent. All experiments conducted in these studies also conformed strictly to guidelines outlined for hESC research by the National Academy of Sciences. All experiments conducted in these studies that involved mouse procedures (including subcutaneous/intra-muscular injections of NOG SCID mice for teratoma analysis) were reviewed and approved by the Johns Hopkins University Animal Care and Use committee (IACUC). These reviews included considerations for ethical sacrifice, human housing, and appropriate measures to minimize animal discomfort.

### Cell Culture

All tissue culture reagents were purchased from Invitrogen (Carlsbad, CA) unless otherwise stated. Primary murine embryonic fibroblasts (MEF) were derived in our lab from E13.5 CF1 x DR4 F1 murine embryos as previously described [5], irradiated at 5000 rad, and used at no later than passage 3. All protocols for all mouse procedures, including the preparation of MEF were approved by the Johns Hopkins University IACUC, as outlined above. MEF, hESC and hiPSC cultures were maintained at 37°C, 5% CO_2_ and 85% relative humidity. Medium was changed daily on hESC and established hiPSC cultures. Pluripotent stem cells were maintained on irradiated mouse embryonic fibroblasts (MEFs) in DMEM/F12 (Invitrogen) medium supplemented with 20% Knockout Serum Replacer (KOSR; Invitrogen), 0.1 mM MEM non-essential amino acids (GIBCO), 0.1 mM β-mercaptoethanol (Sigma) and 4 ng ml^−1^ FGF2 (R&D systems, Minneapolis, MN).

Purified (>95%) human CD34^+^ progenitors from neonatal CB, adult bone marrow (BM), and 20–22 week-old fetal liver (FL) were obtained from pooled donors, and purchased from ALLCELLS (Emeryville, CA) or Lonza, (Walkersville, MD). Human mesenchymal bone marrow stromal cells (BMSCs) (Lonza) were cultured in complete MSC medium (Lonza). Keratinocytes were derived from a normal adult donor, with modified methods as described [53] and cultured in a T175 flask coated with EpiLife Coating Matrix and EpiLife Medium with Supplement S7. Fetal fibroblasts harboring the sickle cell mutation (Cat# GM02340), and 56-year old normal female adult skin fibroblasts (Cat# AG07714) were obtained from the Coriell Institute Cell Repository (Camden, NJ), and cultured in DMEM supplemented with 10% heat-inactivated fetal bovine serum (FBS, HyClone, Thermo Scientific, Waltham, MA), 1X MEM non-essential amino acids, 0.1 mM β-mercaptoethanol, 1 mM L-glutamine and 0.5% penicillin/streptomycin. Keratinocytes and fibroblasts were used at low passages, and freshly passaged 2 to 3 days before nucleofections.

### Cell Cycle Analysis

Cell cycle status of fibroblasts or CB cells in the presence of hematopoietic GFs (FTK: Flt3L, TPO, kit ligand (SCF)) hematopoietic GFs was determined by EdU incorporation following co-culture for 72 hours with and without BMSC stromal layers. Prior to +/− BMSC culture, CB samples were either mock-nucleofected, or nucloefected with 4F or 7F plasmids on day 0, as described below. CD34^+^ cells were incubated with EdU 10 uM for 4 hours in FTK medium on Day 0, or 72 hours following nucleofection. Cells were stained with the Click-IT EdU AlexaFluor488 flow kit (Invitrogen, Carlsbad, CA) according to manufacturers instructions, and analyzed on a BD FACScalibur flow cytometer (BD Biosciences, San Jose, CA).

### Generation of Episomal hiPSC

#### Plasmids

The episomal EBNA-based pCEP4 (Invitrogen, Carlsbad, CA) vectors pEP4 EO2S EN2L (*OCT4, SOX2, NANOG, LIN28*), pEP4 EO2S ET2K (*OCT4, SOX2, SV40LT, KLF4*), pEP4 EO2S EM2K (*OCT4, SOX2, MYC, KLF4*), pEP4 EO2S EN2K (*OCT4, SOX2, NANOG, KLF4*), and pEP4-M2L (*MYC, LIN28*) were obtained from Addgene (Cambridge, MA). Plasmids were propagated in TOP10 *E.* coli (Invitrogen) and purified with QIAGEN plasmid Maxi kits. Ratios of (1∶1∶1) of each plasmid pCEP4-EO2S-EN2L, pCEP4-EO2S-ET2K, and pCEP4-EO2S-EM2K were mixed as the seven-factor (7F) SOKMNLT “Combo 6″ [1]. Plasmid pEP4 EO2S EM2K was used singularly for four-factor (4F) SOKM factor nucleofections.

#### Generation of non-integrated fibroblast-iPSC and keratinocyte-iPSC

Fetal fibroblasts (FFB) cells were passaged two to three days prior to nucleofection. Cells were trypsinized, counted, and 1×10^6^ cells were resuspended in 100 µL of nucleofector solution (VCA-1001, Lonza), and a total of 8 µg of the three 7F episomal plasmids, or 4F single episome. The mixture of DNA/cells solution was nucleofected with program U-020 with an AMAXA II nucleofector device. Adult fibroblasts were obtained from a normal 56 year-old donor, and nucleofected in NHDF nucleofector solution (VPD-1001) with 6 µg 7F plasmid mixture per 1×10^6^ cells using program U023. After nucleofection of either fetal or adult fibroblasts, 500 µL of pre-warmed fibroblast medium was added into the cuvette, and the cells were removed immediately and transferred into three 10 cm plates precultured with irradiated MEF. After 4–6 hours incubation the cells were collected, and fresh fibroblast medium was replaced onto the same MEF cultures (P_0_). After 72 hours (day 3), the fibroblast medium was replaced with hESC medium containing 40 ng mL^−1^ FGF2. Adult keratinocytes were similarly prepared and 1×10^6^ cells were nucleofected using Human Keratinocyte Nucleofector Kit (VPD-1002, Lonza, Walkersville, MD). Keratinocytes were resuspended in 100 µL of Keratinocyte nucleofector solution with of 6 µg of episomal plasmid DNA mixtures and nucleofected with program T-024. After treatment, 500 µL of pre-warmed medium was added into the cuvette, and cells were removed immediately and plated into pre-warmed EpiLife medium with 10% FBS onto MEF feeders. After 4–6 hours incubation, the medium was changed with fresh EpiLife medium. After 72 hours (Day 3), the medium was replaced with hESC medium with 40 ng mL^−1^ FGF2. For both fibroblast and keratinocytes cultures, cells were fed with MEF-condition medium (CM) containing 40 ng mL^−1^ FGF2 after Day 10, and passaged onto fresh MEF layers after 3 weeks (P_1_).

#### Generation of non-integrated CB-iPSC

The method of generation of BMSC-primed CB-iPSC, and the derivation and characterization of non-integrated episomal CB-iPSC clones 6.2, 6.11, 6.13, and 19.11 were recently described [5]. A schematic for quantitatively evaluating comparative reprogramming efficiencies is summarized in **Fig.** **S1**. On Day -3 of the reprogramming protocol, 0.5×10^6^–1.0×10^6^ purified human CD34^+^ progenitors from fetal liver, neonatal CB, adult mobilized peripheral blood, and adult bone marrow were thawed, expanded in two ml Stem Span-SFEM medium (StemCell Technologies, Vancouver, BC), and supplemented with FLT3L (100 ng mL^−1^), and TPO (10 ng mL^−1^), SCF/Kit ligand (100 ng mL^−1^), (FTK) (R&D Systems, Minneapolis, MN). All reprogramming culture steps were conducted in tissue culture plates that were tightly wrapped in Saran wrap for induction of hypoxic conditions. After three days (on day 0), cells were collected by centrifugation (200 *g*, 10 min) and counted. 0.5×10^6^–1.0×10^6^ CD34 progenitors were nucleofected with 6 µg total of 4F or combined 7F plasmid DNA (combination 6 or 19, as above) using the AMAXA II nucleofector device (Lonza), program U-008, and 100 µL CD34^+^ nucleofector solution VPA-1003 (Lonza). Following nucleofection, 500 µL of pre-warmed medium was added into the cuvette, and cells were replated immediately into one mL pre-warmed RPMI 1640 medium with 10% FBS in a 12 well plate. After 4–6 hours incubation in RPMI/10% FBS, nucleofected CD34^+^ cells were collected and replated onto Retronectin (Takara Bio, Madison, WI) -coated (10 µg mL^−1^) 6-well plates seeded with confluent, irradiated (2000 cGy) human mesenchymal bone marrow stromal cell (BMSC) feeders. Nucleofected Day 0 CB progenitors were expanded in these BMSC cocultures in SFEM supplemented with 100 ng mL-1 FLT3L, 50 ng mL^−1^ TPO, and 100 ng mL^−1^ SCF (FTK GFs). Three days later (Day 3), CB or CB-BMSC cultures were harvested enzymatically, and single viable CB cells were counted, and 300–20,000 cells were replated onto irradiated MEF feeder plates in 2 mL SFEM containing FTK GFs, as above. On Day 4, two mL of hESC medium containing 40 ng mL^−1^ FGF2 was added to MEF cultures. On Day 6, and every 2 days thereafter, one-half the medium volume in each well was harvested (hemidepletion), and hematopoietic suspension cells were returned into their respective wells with 2 mL fresh hESC medium containing 40 ng mL^−1^ FGF2 (*i.e.,* gradually tapering the concentration of FTK GFs from Day +3). Starting on Day 12, MEF-conditioned medium (CM) supplemented with 40 ng mL^−1^ FGF2 was used for subsequent medium changes. hESC-like colonies emerged with these conditions with CD34^+^ progenitors as early as 7–10 days post-nucleofection.

### Flow Cytometry, Immunocytostaining, Alkaline Phosphatase, and Live TRA-1-81 Surface Antigen Staining

#### Flow cytometry

hESC and hiPSC cultures were dissociated enzymatically, passed through a 40 µm filter to remove cellular debris, and then centrifuged for 5 min at 200 *g*. The cells were gently resuspended in PBS containing 5% FBS, and stained with monoclonal antibodies for 30 min on ice. Antibodies included APC conjugated SSEA4 (R&D Systems), PE Mouse anti-Human TRA-1-60 antigen (BD Biosciences) and PE Mouse anti-Human TRA-1-81 antigen (BD Biosciences). For intracellular OCT3/4, SOX2, and NANOG FACS staining, cells were fixed and permeabilized using FIX & PERM Cell Permeabilization Reagent (Invitrogen), and the cells were stained with anti-human/mouse OCT3/4-PE (R&D Systems), SOX2-PE, or mouse anti human Nanog-PE (BD Biosciences). Cells were washed in 5% FBS/PBS and analyzed using a FACSCalibur instrument (BD Biosciences). Data were analyzed using FLOWJO flow cytometry analysis software (www.flowjo.com).

#### hiPSC colony enumeration by alkaline phosphatase (AP) staining and live TRA-1-81 staining

hiPSC cultures were fixed in 4% paraforaldehyde/PBS for 10 minutes, and washed in 1X PBS and stained with AP substrate in 1 step NPT/BCIP reagent (Sigma) for 10 to 15 min at room temperature. The reactions were stopped after 15 minutes, and wells were washed three times with 1X PBS. Only colonies that stained strongly and within 15 minutes (AP^hi^) were enumerated. In alternate replicate wells, TRA-1-81 StainAlive Dylight 488-conjugated antibody (1∶100; Stemgent, Cambridge, MA) was diluted in hESC medium and directly added into P_0_, and later in P_1_ iPSC cultures. After 30 min, cultures were washed twice with hESC medium, and TRA-1-81 positive colonies were visualized with fluorescence microscopy. Three to five weeks following episomal nucleofections, hESC-like colonies were counted and stained live with TRA-1-81 fluorescent antibodies on the original of P_0_ MEF cultures, and fluorescent colonies were enumerated.

### Reprogramming Efficiency Determinations

The experimental design for determining comparative reprogramming efficiencies of CB, fibroblasts, and keratinocytes is summarized in **Fig.** **S1**. The efficiency of hiPSC generation from single unfractionated or FACS-purified CB populations was determined directly on MEF cultures following 6 days of GF stimulation (which included 3 days of +/− BMSC priming (from Day 0 (4F nucleofection) to Day 3). The experimental details for episomal reprogramming of FACS-purified CD34^+^CD38^+^ and CD34^+^CD38^lo^ hematopoietic populations are described below. A schematic that summarizes the reprogramming strategy of FACS-purified hematopoietic populations, including the enrichment of lineage-committed myeloid progenitors that co-expressed reprogramming transgenes and a GFP reporter is outlined in **Fig.** **2**.

Reprogramming efficiencies were determined 3–5 weeks following episomal nucleofections on the original (P_0_) cultures (without additional subsequent MEF passages) based on criteria for identifying TRA^+^NANOG^+^Alkaline phosphatase (AP)^+^ ‘Type III’ hiPSC [43] (see **Fig.** **S3**). The number of Type III colonies that emerged were enumerated (per single input cells plated on day 3) and defined as those colonies possessing well-defined hESC-like borders, compact morphology, large nuclei, rapid and strong high alkaline phosphatase (AP^hi^) staining (Sigma-Aldrich, St. Louis, MO) and positivity for live TRA-1-81 antibody immunostaining (StainAlive™DyLite™488 Mouse anti-Human TRA-1-81 antibody, Stemgent). Since 50–80% of all CB-iPSC colonies acquired TRA and NANOG positivity by 3–5 weeks (**Figs. 2a** **,** **7a**), we found that the rigorous enumeration of AP^hi^-staining CB-iPSC with morphologies of hESC correlated directly, and was quantitatlvely comparable to enumeration with live TRA-1-81 staining of the same colonies (see **Fig.** **S3**). Thus, the results of rapid AP^hi^ staining of these ‘hESC-like’ colonies are presented in most cases for clarity. Details of all staining methods are provided below.

Passage 0 (P_0_) hiPSC cultures were fed with MEF conditioned medium (CM) supplemented with 40 ng mL^−1^ bFGF after 12 days, and this was continued until AP assays or live TRA-1-81 stainings were performed 3–5 weeks following original nucleofections (See **Fig.** **S1**). Individual hESC-like subclones were also manually picked from P_0_ (CB-iPSC) or P_1_ (Fib-iPSC; Ker-iPSC) cultures for expansion and further characterizations.

To determine the completion of reprogramming in whole bulk populations of these emerging hiPSC cultures, we also analyzed P_0_ reprogrammed cultures by FACS for surface SSEA4, surface TRA-1-81, and intra-cellular NANOG expressions 3–5 weeks following initial MEF platings. Bulk cultures were stained with surface antibodies (BD Biosciences, San Jose, CA) for pluripotency markers (SSEA4-APC, TRA-1-60-PE, TRA-1-81-PE) or hematopoietic markers (CD34-PE, CD45-APC, CD34-APC, CD33-PE, CD13-PE). Cells were fixed and permeablized with Fix and Perm kit (Invitrogen) for intracellular NANOG-PE FACS analysis.

### Reprogramming Efficiency Determinations of FACS-purified Hematopoietic Populations

#### Episomal reprogramming of FACS-purified CD34^+^CD38^+^ and CD34^+^CD38^lo^ hematopoietic populations

A schematic that summarizes the reprogramming strategy of FACS-purified populations is included in **Fig.** **2a**. Highly purified (>96%) CD34^+^CD45^+^ CB cells were obtained commercially (AllCells), and thawed according to manufacturer’s instructions. CD34^+^ CB cells were cultured overnight initially (day -3; **Fig.** **S1**) in Stem Span-SFEM medium (StemCell Technologies, Vancouver, BC) supplemented with FLT3L (100 ng mL^−1^), and TPO (10 ng mL^−1^), and SCF/Kit ligand (100 ng mL^−1^), (FTK) (R&D Systems, Minneapolis, MN) overnight. The next morning (day -2.5), viable cells were collected for FACS purification in Stem Span-SFEM medium and centrifuged in 200 g, 5 min. CD34^+^ cells were stained with mAb CD38-APC (BD Biosciences) for 30 min on ice. FACS gates for both CD38^high^ and CD38^low^ -expressing cells (19.4±7.39%, and 19.73±7.24% respectively, n = 3) were identified (e.g., see Fig. S11b), and purified CD34^+^ populations were collected in SFEM medium containing FTK GF (as above), and cultured an additional two days (until day 0; **Fig.** **S1**). On day 0, FACS-purified CD34^+^CD38^hi^ and CD34^+^CD38^low^ populations were nucleofected with a single episome expressing 4F (pEP4 EO2S EM2K; see Suppl Methods), and cultured further in GF +/−BMSC co-culture, exactly as described above, for an additional 3 days (**Fig.** **S1**), Single CB cells from each purified population were plated on MEF on day 3 for subsequent reprogramming efficiency determinations, exactly as described above for unsorted CB cells).

#### FACS-purification of lineage-committed myeloid progenitors exclusively expressing reprogramming episomes via GFP co-expression

A schematic that summarizes the reprogramming strategy of FACS-purified populations enriched for expression of reprogramming episomes is included in **Fig.** **2a**. For these experiments, highly purified (>96%) CD34^+^CD45^+^ CB cells were received within 24 hours of neonatal harvest from AllCells (catalog number: CB005). On the same day (day -3 of the reprogramming protocol; **Fig.** **S1**), CB cells were plated in 2 mL of Stem Span-SFEM medium (StemCell Technologies, Vancouver, BC) supplemented with hematopoietic GFs: FLT3L (100 ng mL^−1^), TPO (10 ng mL^−1^), and SCF/Kit ligand (100 ng mL^−1^; all R&D Systems, Minneapolis, MN). Culture plates were tightly wrapped in Saran wrap to create hypoxic cultures. On day 0, GF-activated myeloid progenitors were collected in Stem Span-SFEM medium, and centrifuged at 200 g for 5 min. 0.5×10^6^ CD34 progenitors were nucleofected with 6 µg of 4F-plasmid DNA (pEP4 EO2S EM2K) and 2–3 µg of pEP4-EF1a-eGFP (with same vector backbone and promoter as the 4F episomal construct) using the AMAXA II nucleofector device (Lonza). Program U-008, and 100 µL CD34^+^ nucleofector solution VPA-1003 (Lonza) was employed. Following nucleofection, 500 µL of pre-warmed medium was added into the cuvette, and cells were replated immediately into one mL pre-warmed RPMI 1640 medium with 10% FBS in a 12 well plate. After 4–6 hours, nucleofected CB cells were collected, centrifuged, resuspended in Stem Span-SFEM medium with FLT3L, TPO, SCF/Kit ligand (100, 50, 100 ng mL^−1^, respectively), and plated onto Retronectin (Takara Bio, Madison, WI) -coated (10 µg mL^−1^) 12-well plates, or onto irradiated (2000 cGy) BMSC feeder layers that were similarly pre-coated with Retronectin. Three days later (day 3), CB cells were harvested, and stained with CD34-PE (BD BioSciences) antibody for 20 min on ice. BMSC were easily distinguished from CB cells by forward scatter and side scatter gates, and excluded for cell sorting. Three populations of day 3 CB cells were purified based on GFP expression: **GFP^-^**CD45^+^ (non-transgene-expressing cells), **GFP^+^**CD34^+^CD45^+^, and **GFP^+^**CD34^−^CD45^+^ expression (transgene-expressing cells). These sorted GFP^+^ and GFP^-^ CB populations were plated onto MEF on day 3, and reprogramming efficiencies determined, as above.

### Expression Microarrays, Bioinformatics Data, and Gene Set Enrichment Analysis (GSEA)

The NIH Gene Expression Omnibus has issued the accession number GSE35029 for our deposited microarray data related to this manuscript. The GEO-supplied link to the deposited data is:

http://www.ncbi.nlm.nih.gov/geo/query/acc.cgi?token=trmxdeemyoomcfq&acc=GSE35029

#### Collection and preparation of cell samples for expression microarrays

Bulk reprogrammed cultures were collected from CB-BMSC (on day 3) or reprogrammed CB-MEF co-cultures (on day 23) and filtered through a 40 µm cell-strainer. Samples were purified by FACS sorting based on viability (day 23 samples), or surface CD45^+^ expression (for day 3 samples). FACS-purified cells were kept on ice until centrifuged and snap frozen in liquid nitrogen for RNA purification and subsequent Illumina gene array analysis. All hESC/iPSC lines were confirmed to be >98% SSEA4^+^TRA-1-60^+^TRA-1-81^+^ by FACS prior to harvesting cell pellets for RNA to be used in qRT-PCR or Illumina gene microarrays. All pluripotent stem cell lines were passaged from MEF onto Matrigel and expanded with MEF-conditioned medium (CM) for one passage prior to harvesting cells for expression studies to assure removal of nonviable, irradiated MEF.

#### Gene expression microarrays

Details for collection of cell samples for microarrays are provided above. Human HT-12 Expression BeadChip arrays (Illumina, San Diego, CA) were used for microarray hybridizations to examine the global gene expression of hESC, hiPSC, and starting populations (CD34^+^ progenitors, keratinocytes, and fibroblasts). Each array on the HumanHT-12 Expression BeadChip array targeted more than 25,000 annotated genes with more than 48,000 probes derived from the National Center for Biotechnology Information Reference Sequence (NCBI) RefSeq (Build 36.2, Rel 22) and the UniGene (Build 199) databases. Total RNA was prepared as described in the RNeasy Mini Kit (QIAGEN) with on-column DNase I digestion. All samples were processed at the Sidney Kimmel Comprehensive Cancer Center Microarray Core Facility at Johns Hopkins University, Baltimore. Briefly, 200 ng total RNA from each sample was amplified and labeled using the Illumina TotalPrep RNA Amplification Kit, AMIL1791 (Ambion, Austin, TX) as described in the manufacturer’s instruction manual. All arrays were hybridized at 58°C for 16–20 hours followed by wash and stain procedures according to the Whole-Genome Gene Expression Direct Hybridization Assay Guide (Illumina, San Diego, CA). Fluorescent signals were obtained by scanning with the iScan System and data were extracted with Gene Expression Module 1.0.6 in GenomeStudio 1.0.2 and signal intensities from multiple chips were normalized without background subtraction.

#### Bioinformatics data analysis

Gene expression data from the Human HT-12 arrays, described above, were analyzed with the Partek Genomics Suite (Partek Inc., St. Louis, MO) and Spotfire DecisionSite for Functional Genomics™ (TIBCO Software Inc., Somerville, MA) platforms. The scanned fluorescent signal data were quantile normalized in Illumina Bead Studio to allow cross array comparison, and were then imported into Partek where they were first log2 transformed for analysis. For heatmaps presented, these expression values were mean-normalized to better demonstrate how gene expression differed across the examined cell types. In mean normalization, each gene’s mean log2 signal value is determined for all cell types and then subtracted (division in log space) from each cell type’s value for that gene. The normalized values underwent unsupervised hierarchical clustering in Spotfire (Euclidean distance algorithms) to compare cell types’ gene expression in a heatmap-dendrogram wherever indicated (color spectrum indicates where lower = blue; higher = red). The R^2^ values shown are the square of the Pearson R correlation coefficient between the two cell types’ correlation, where higher value indicates greater correlation (all R values were positive). Partek software was used to compare the mean normalized log2 signal values of pluripotency-associated gene modules (e.g. ESC core, MYC, PRC1, PRC2, Core) in box and whisker plots, and Spotfire to determine the Pearson R correlation coefficient (PCC) between cell types’ log2 expression values.

#### Gene Set Enrichment Analysis (GSEA)

Significantly expressed gene sets were determined from normalized Illumina array data using the GSEA computational method [45] (http://www.broadinstitute.org/gsea). The GSEA method determines whether an *a priori* defined set of genes shows statistically significant, concordant expression differences between two biological states. GSEA was performed on day 0 CB and day 3 (+/− BMSC-primed) CB microarray samples, as described above. These curated sets of genes were analyzed using GSEAP v2.07 (http://www.broad.mit.edu/gsea) software and the MSigDB v. 3.0 CP (canonical pathways) gene sets, with an FDR <0.05 chosen as a threshold for significance.

### Polymerase Chain Reaction (PCR)

#### Reverse Transcriptase (RT) and genomic PCR

RT-PCR analysis for transgene expression and *EBNA1* vector backbone were performed with primers as described in Yu *et* *al*., 2009, and Burridge et al, 2011 [1], [5]. Briefly, total RNA was extracted from passage 11 CB-iPSC clones, negative control passage 48 H9 hESC, and positive control “bulk” (passage 2) early CB-iPSC that were nucleofected with episomal vectors (∼14–21 days old) using the RNeasy Mini Kit (QIAGEN). cDNA was generated from each sample using SuperScript-First Strand Synthesis (Invitrogen), and PCR reactions were performed with Pfx DNA polymerase (Invitrogen) using the protocol described previously. PCR products were analyzed on 2% agarose quick gels (Invitrogen). Genomic and episomal DNA were extracted from passage 11 CB-iPSC, negative control H9 hESC, and positive control bulk CB pre-iPSC using DNeasy Blood & Tissue Kit (QIAGEN). Genomic PCR reactions were performed with Pfx DNA polymerase as described in Yu *et* *al*., 2009 [1]. PCR products were analyzed on 2% agarose gels.

#### quantitative Real-Time RT-PCR (qRT-PCR)

Total RNA from all hiPSC/hESC or donor cell samples was prepared using the RNeasy Mini Kit with on-column DNase I digestion (QIAGEN). First-strand cDNA was reverse transcribed with oligo-dT using SuperScript First-Strand (Invitrogen). qRT-PCR was performed using iQ SYBR-Green (BioRad, Hercules, CA) or Power SYBR PCR Mastermix (Applied Biosystems, Foster City, CA) and ABI thermal cycler and software. Human-gene specific PCR amplicons of 90–300 bp (see PCR Primers ref [53]) were designed with PRIMER 3.0 software (http://frodo.wi.mit.edu/primer3/), and all primers were optimized for the following conditions: initial denaturation for 5 min at 95°C; 45 cycles of 95°C 15 sec, 60°C 30 sec, 68°C 30 sec. Transcripts of target genes and beta actin controls for each cDNA sample were amplified in triplicates/quadruplicates. All qRT-PCR reactions were confirmed for specificity of a single PCR product by analysis on 4% agarose quick gels. Relative qRT-PCR analysis using the 2^-ΔΔCT^ method was performed using cycle threshold (C_T_) normalized to beta actin, as described [54], [55]. Fold change expression of actin-normalized CB-iPSC clones was compared to control H9 hESC. For the analysis of endogenous gene expression of nucleofection target cells for iPSC formation, HSC GF-activated CB (AllCells) were thawed, expanded for 3 days in Flt3L (100 ng/ml), TPO (10 ng/ml) and SCF (100 ng/ml), and used for RNA and cDNA preparation followed by qRT-PCR analysis relative to control H9 hESC, as described above. PCR primers used in these studies to evaluate transgenic (episomal) and endogenously expressed pluripotency genes were previously described [5], [53].

### Teratoma Assays

Passage number between 10 to 15 hiPSC lines were passaged from MEF onto Matrigel cultures and expanded with MEF-conditioned medium (CM) prior to harvest and teratoma injections. Briefly, hiPSC were grown to 60–80% confluency on Matrigel/CM, harvested as clumps with collagenase IV (Invitrogen) or cell scrapers, resuspended in a mixture of hESC medium and Matrigel (BD Biosciences) at a ratio of 1∶1, and ∼10^7^ cells were injected intramuscularly (hind leg) or subcutaneously into immunodeficient NOG SCID mice (approximately one 6-well plate per mouse). After six to twelve weeks, teratomas were dissected, fixed in 4% paraformaldehyde, embedded in paraffin, and stained with hematoxylin and eosin.

### Karyotypes of Pluripotent Stem Cell Lines

Karyotyping was performed by high resolution G-banding at the Johns Hopkins School of Medicine Cytogenetics Core.

### Proteomic Studies of CB-BMSC Co-culture Supernatants

Replicate media supernatants were harvested from Day 3 CB cells that had been co-cultured with (+) or without (−) irradiated BMSC layers for 3 days in SFEM-FTK and Retronectin in conditions exactly as for reprogramming experiments. Supernatants were frozen at −80C, and later analyzed by high-throughput antibody arrays that probed for >300 human cytokines (RayBio Custom G-series Human Cytokine Antibody, G-series glass chip, RayBiotech, Norcross, GA). Raw intensity values from array analysis were normalized to positive controls and background subtracted. Expression of molecules was normalized and ranked based on the ratio of their expression in BMSC-conditioned *vs*. non-conditioned media.

## Supporting Information

Figure S1 **Summary of experimental design for determining comparative episomal reprogramming efficiencies of human somatic target cells.** Details of reprogramming efficiency assays are described in Methods. Reprogramming efficiencies of (**a**) GF-activated and +/− BMSC-primed CB progenitors, or (**b**) fetal and adult fibroblast (FFB; AdFib) and adult keratinocyte (Ker) populations were determined on MEF cultures (plated on day +3) following plasmid nucleofections on Day 0 with a single plasmid expressing four (4F) transgenes, or three plasmids expressing seven (7F) episomal transgenes. Day 0 nucleofected CB cells were briefly co-cultured with (or without) irradiated adult BMSC stromal layers and continued hematopoietic GFs (Flt3L, TPO, Kit ligand-SCF (FTK)) from Day 0 to Day +3. Single cells were subsequently re-plated on irradiated MEF and cultured in hESC medium supplemented with 40 ng/ml bFGF. Reprogramming efficiencies of emerging hESC-like (CB-iPSC) colonies were determined in these original (P_0_) MEF cultures at 3–5 weeks post initial nucleofections. After 12 days on MEF, cultures were fed daily with MEF-conditioned medium (CM) supplemented with 40 ng/ml bFGF. Reprogramming efficiencies for somatic targets were determined via two independent methods in averaged triplicate-quadruplicate cultures for each experiment: 1) counting the number of colonies that emerged per single cells plated on replicate P_0_ MEF cultures at day 21–25 that had hESC-like morphology (as defined by compact hESC characteristics with large nuclei and nucleoli and that had high alkaline phosphatase staining (AP^+^; AlkPhos^hi^). Alternatively, 2) hESC-like colonies, as defined above, and that were positive for live Tra-1-81 surface staining were enumerated in replicate cultures. In many experiments, both assays were done in parallel on the same cultures (live TRA staining, followed by fixation and AP stating (see Fig. S2). hESC-like/AP^+^/Tra-1-81^+^ colonies emerged from nucleofected CB as early as 7–21 days post-nucleofection. Both efficiency assays gave comparable results and AP^+^ hESC-like colony assays are generally presented in this manuscript. Additionally, since a large majority of BMSC-primed CB cells populations converted to hESC-like colonies, in some experiments, the completion of reprogramming in whole populations of actively-reprogramming cells was also estimated via FACS expression of intracellular NANOG staining, and surface TRA-1-81 and SSEA4 staining of whole, bulk cultures. Unlike 4F or 7F-nucleofected CB, 7F-nucleofected keratinocytes and adult or fetal fibroblast cells never produced hESC-like colonies on initial P_0_ MEF and CM cultures at 3–5 weeks. 7F episomal fibroblast-iPSC, and keratinocyte-iPSC colonies emerged rarely for these donor types, and did so only after subsequent passage (P_1_) on fresh MEF at 3–4 weeks. Also, keratinocytes and fibroblasts never produced 4F episomal hiPSC. Thus, bulk fibroblast and keratinocyte P_0_ reprogramming cultures (in CM with 40 ng/ml bFGF) were passaged after 4 weeks with 1 mg mL^−1^ of collagenase IV onto fresh irradiated MEF layers (P_1_) at a ratio of 1∶1–1∶6) for the expansion of slowly reprogramming precursors. Estimated efficiencies for fibroblast-iPSC and keratinocyte-iPSC were determined on these secondary P_1_ MEF cultures ∼1 week later with AP staining and FACS assays, as above.
(TIF)

Figure S2 **Brief co-culture of growth factor-activated Day 0 CB cells with BMSC preserved their multipotent hematopoietic progenitor frequencies.** Brief co-culture of GF-activated CB cells (Flt3L, SCF, and TPO from Day -3 to Day 3) with irradiated BMSC plus continued GFs for an additional 3 days (from Day 0 to Day 3 of reprogramming protocol; see Fig. S1) increased the frequency of a) Day 3 phenotypic multipotent hematopoietic CD34+CD45+ progenitors and b) Day 3 erythro-myeloid GEMM-CFU. There was enhancement to a lesser extent in mobilized CD34+ peripheral blood progenitors (mPB). CFU colony assays of GF-activated Day 0 CB cells were conducted in semi-solid methylcellulose as previously described [54], [55].
(TIF)

Figure S3 **Correlation of Alkaline Phosphatase (AP) of hESC-like colonies and live TRA-1-81 staining assays for measuring non-integrated reprogramming efficiencies of human hematopoietic progenitors.** Efficiencies for the number of hESC-like colonies that emerged per single cells input into Day 3 MEF cultures was determined in parallel 3–5 weeks following 4F and 7F nucleofections with two independent methods: 1) AP^+^ staining and 2) live TRA-1-81 staining (Dylight 488, green). Details of assays are described in Methods. Shown here are photomicrographs of a representative early P_0_ hESC-like colony which emerged 14 days following MEF plating of single 4F-nucleofected Day 0 CB progenitor cells, as described in text. (**a**) hESC-like colony after live TRA-1-81 immunostaining, (**b**) Same ESC-like colony, with merged phase contrast and live immunofluorescent TRA-1-81 staining. (**c**) Same hESC-like colony following fixation and AP staining (purple). Note that live TRA-1-81 staining (which indicates conversion to a completed Type III reprogrammed state) is more heterogeneous in early P_0_ colonies, and appeared with slower kinetics than the rapid dark AP staining which was dark and homogenously stained. Scale bars = 100 µ (microns).
(TIF)

Figure S4 **Nucleofection of large episomal plasmids into Day 0 CB cells is inefficient.** Gene transfer efficiency of Day 0 CB progenitors, adult human fibroblasts, or 293T embryonic kidney carcinoma cells was determined by GFP reporter expression with either a 3 kb CMV-GFP plasmid (pMAX-CMV-GFP; AMAXA kit) or a ∼15 kb EBNA-based GFP episome (pCEP4-EF1-GFP) of similar size, and with the same promoter and vector backbone as our 4F and 7F reprogramming plasmids. (**a**) Results of averaged experiments for 48 hr GFP expression of Fibs or CB cells nucleofected on Day 0 with 6 µg plasmids per 500,000 cells. Time courses of GFP expression following (**b**) 293T transfections (Lipofectamine 2000), or (**c**) BMSC-primed CB nucleofections of each indicated plasmid. These experiments revealed that large pCEP4 EBNA-based episomes were excellent expression vectors via transfection, but possessed limiting nucleofection gene transfer efficiency, likely due to their large sizes. A second pulse of plasmid (**c**, 2nd nucl) was nucleofected on day 3 in some experiments, but did not dramatically improve the low gene transfer efficiency of the original pulse (1st nucl).
(TIF)

Figure S5 **Kinetics of emergence and stability of undifferentiated colony morphology of early passage episomal hiPSC clones from 4F cord blood and 7F fibroblast -nucleofected cells.** TRA-1-81+ hESC-like colonies emerged rapidly with 4F-nucleofected BMSC-primed CD34^+^ CB cells (as early as 7–14 days in initial P_0_ MEF co-cultures), and at significantly higher efficiencies than without BMSC co-culture. Additionally, unlike episomal fetal fibroblast-iPSC or non-BMSC-primed CB-iPSC colonies (not shown), the majority (>90%) of BMSC-primed CB-iPSC clones maintained a stable undifferentiated hESC-like morphology with minimal spontaneous differentiation that permitted manual picking and expansion with minimal effort. Shown in (**a**) is typical morphology of an emerging 4F CB-iPSC clone at day 7, 9,14 and 21 with (**b**) live TRA-1-81 staining), as well as following expansion at (**c**) passage 2 (P2). Note the stable, well-circumscribed borders of the P2 episomal CB-iPSC colony with minimal differentiation (white arrow). In contrast, although abnormal granulated colonies (**d**) often emerged for 7F-nucleofected fibroblasts at P_0_, colonies with hESC-like morphology did not, and only emerged with slow kinetics after 1–2 MEF passages, and with unstable spontaneous differentiation that required extensive subcloning. Shown in (**e**) is a representative 7F fib-iPSC colony at P2 with typical spontaneously differentiating borders (red arrow).
(TIF)

Figure S6 **Generation of non-integrated 7F episomal hiPSC from fibroblasts with EBNA1-based episomal plasmids.** 22 week-old lung fetal fibroblasts carrying the homozygous sickle cell disease mutation were obtained from the Coriell Cell Repository (GM02340), and used to generate nonviral human fetal fibroblast-derived SSEA4^+^TRA-1-60^+^ hiPSC (**a,b**; at low passage) with seven episomal factors, as described in the text, that (**c**) demonstrated differentiation to all three germ layers in NOG teratoma assays. Shown are H&E stains of teratoma sections from SCD-hiPSC demonstrating elements of ectoderm (neural rosettes, retinal pigmented epithelium, endoderm (glandular epithelium), and mesoderm (bone, muscle). Shown also (**d**) are genomic PCR and RT-PCR assays confirming the lack of integration and expression of transgenic episomal constructs. Details for these transgene-specific PCRs were previously described [5].
(TIF)

Figure S7 **Generation of non-integrated 7F episomal hiPSC from normal adult hair follicle-derived keratinocytes.** Keratinocyte lineage cells were confirmed by CD49f (alpha-integrin)-positive, CD71-low cells after expansion from a single plucked hair (**a**; left panel) of a normal adult donor, using methods as described [53]. ∼2×10^6^ cell were nucleofected with “Combo 6″, re-suspended in fresh culture medium, and then transferred onto gelatinized PMEF plates. After 48–72 hours, media was replaced with hESC medium or CM for three weeks (P_0_), followed by replating onto fresh PMEF (P_1_). (**b**) Colonies with hESC-like morphology, and expressing pluripotency markers (e.g., SSEA4, Tra-1-60/81, CD90, OCT4, NANOG, SOX2), emerged with rare efficiencies (see **Fig.** **1**) 1–2 weeks following P_1_ culture of nucleofected cells. Nonviral iPSC clones derived from keratinocytes. KER-iPSC were further subcloned, and confirmed for lack of integrated episomal sequences by RT-PCR (**c**) of pluripotency transgenes, expanded for frozen stocks, and confirmed for pluripotency by tri-lineage cystic teratoma formation assay (not shown).
(TIF)

Figure S8 **Generation of non-integrated 4F and 7F episomal CB-iPSC lines.** (**a,b**) Representative colony morphology (**a**) and FACS staining (**b**) of SSEA4, TRA-1-81, and CD90 surface pluripotency markers from CB-iPSC lines generated as described in Methods, with four (4F) or seven (7F) episomal reprogramming factors. Full characterizations, including Southern blots, genomic PCR, and RT-PCR for validations of vector and transgene-free status of CB-iPSC lines that we evaluated by expression microarrays in **Fig.** **S10** *(i.e.,* clones 6.2, 6.11, 6.13, and 19.11) were previously reported [5]. (**c**) H&E stains of cystic teratomas obtained from a representative CB-iPSC line 6–8 weeks following injection into NOD/SCID mice illustrate well-differentiated cell lineages of all three germ layers, including regions containing neural rosettes, pigmented retinal epithelium, glandular epithelium, fetal intestinal structures, cartilage, striated muscle, and hyalinized bone. Ectodermal structures (Ect): neural rosettes (left); retinal pigmented epithelium (right); Endodermal structures (End): glandular epithelium (left); developing gut loop (right); Mesodermal structures (M): cartilage (left), bone/muscle (right). All CB-iPSC lines described in this manuscript formed similar tri-lineage cystic teratomas. Analysis of histological sections also demonstrated that these teratomas were completely devoid of foci of malignant transformation. Scale bars = 100 µ (microns).
(TIF)

Figure S9 **Episomal integration and karyotypes of non-integrated 4F and 7F episomal hiPSC derived from CD34+ CB and FL progenitors.** (**a,b**) 4F and 7F CB-iPSC and FL-iPSC were assayed by transgene-specific genomic PCR at indicated passages exactly as previously described [5] for episomal sequences. Bulk P1 4F CB-iPSC cultures serve as a positive control. (**c**) G-band karyotyping on representative 4F and 7F CB-iPSC lines. Experimental details for genomic PCR and karyotyping are provided in Methods.
(TIF)

Figure S10 **Genome-wide expression studies of non-integrated hiPSC lines revealed that stromal-primed low passage CB-iPSC lines possessed transcriptional signatures that were highly akin to hESC at low passage.** To examine the quality of nonintegrated reprogramming achieved, low passage hiPSC clones were derived from fetal fibroblasts (**Fig. S5**), keratinocytes (**Fig. S6**), as well as stromal-primed CB donors (**Fig. S7, S8**). And their transcriptional signatures were evaluated by Illumina microarray expression analysis. Non-integrated hiPSC were generated with the same 7F episomal constructs, and global gene expressions were compared. All non-integrated hiPSC lines were confirmed to be free of transgene and vector sequences by Southern blotting, genomic PCR, and RT-PCR at early passage (p9–12), as previously described [5] and (**Figs. S5, S6, S7**). Levels of pluripotency markers SSEA4, TRA-1-60, TRA-1-81, OCT4, and NANOG proteins for all hiPSC assayed were found comparable to control hESC. All non-integrated iPSC lines were also tested for their ability to form, well-differentiated tri-lineage cystic teratomas in NOG-SCID mice demonstrating their *bona fide* pluripotency. We determined the expression signatures of these non-integrated hiPSC clones with Illumina microarrays, and also included previously described lentiviral hiPSC lines IMR90-1 and IMR90-2 [56] and H9 hESC as controls. An unsupervised hierarchical clustering of global expression (37,839 genes) from all starting populations and cell lines was computed. Global gene expression samples of episomal lines were evaluated at the earliest passage possible (P_11–14_). H9 hESC (P_51_), episomal CB-iPSC^5^ clones 6.2, 6.11, 6.13, (P_14_), 19.11, (P_11_), nonviral keratinocyte-iPSC clones: KA.1, KA.3 (P_13_); episomal fetal fibroblast-iPSC: F.1, F.6 (P_14_); viral fibroblast-iPSC clones: IMR1 (P_66_), IMR4 (P_64_). This dendrogram represents the unsupervised hierarchical clustering of signal values from all 37,839 genes represented on the Illumina microarray for all cell types examined. Low passage (P_11–14_) non-integrated CB-iPSC samples (n = 4) had global expression profiles that highly correlated to hESC (Pearson coefficients R^2^ = 0.98). Fibroblast-iPSC and keratinocyte-iPSC had Pearson coefficients of R^2^ = 0.96 relative to hESC. Collectively, these studies revealed that 1) CD34^+^ progenitor populations (FL, CB, BM, mPB) were transcriptionally more akin to pluripotent stem cells as a group, 2) low passage (P_11–14_) non-integrated CB-iPSC samples (n = 4) had global expression profiles that more faithfully correlated (Pearson coefficients R^2^ = 0.98) with those of control hESC, and 3) stromal-primed reprogramming could generate high quality CB-iPSC that resembled hESC at low passages.
(TIF)

Figure S11 **Endogenous expression of known reprogramming factors in parental donor populations.** (**a**) Endogenous expressions of pluripotency-associated factors were determined by qRT-PCR analysis on Day 0 of the reprogramming protocol of GF-activated (FTK) donor cell populations (CD34^+^ fetal liver; FL), CD34^+^ cord blood (CB), GCSF-mobilized peripheral CD34^+^ blood (mPB), adult CD34^+^ bone marrow (BM), adult keratinocytes, and fetal fibroblasts. Shown is the fold change normalized expression levels of each factor relative to expression in control H9 hESC calculated by the 2^−ΔΔCT^ method. Primer sequences are presented in Methods. (**b**) Stem-progenitor (CD34^+^CD38^lo^) and lineage-committed (CD34^+^CD38^+^) populations were FACS-purified from Day -2.5 CB cells, and similarly evaluated for expression of endogenous pluripotency factor transcripts by qRT-PCR.
(TIF)

Table S1 **Lists of stem cell gene modules and epigenetic regulator circuits used in these studies (xls file).**
(XLS)

Table S2 **CB-BMSC secretome studies (xls file).** Rank order analysis of **a**) over-expressed and **b**) under-expressed cytokines and growth factors in Day 3 CB-BMSC (+MSC) or Day 3 CB alone (-MSC) conditioned supernatants, following 3 days of co-culture with SFEM media and Flt3L, TPO, and SCF growth factors. See **Fig. S1** for summary schematic of reprogramming experiments. Conditioned supernatants were frozen at −80C, and later analyzed by high-throughput antibody arrays that probed for >300 human cytokines (RayBio Custom G-series Human Cytokine Antibody, G-series glass chip). Shown are the top most abundant secreted molecules in Day 3 CB-BMSC supernatants (+MSC), listed in descending order by the ratio of their expression to non-BMSC-conditioned Day 3 CB cells (-MSC), with a cut-off ratio of 1.3x-fold. A large fraction of the soluble factors differentially enriched in CB-BMSC supernatants were those known to be directly activated following Toll receptor-NFκB signaling (red) or that are known to regulate Toll receptor, NFκB inflammatory signaling (green).
(XLS)

Table S3 **Gene Specific Enrichment Analysis (GSEA) results of microarray data of purified and 4F-nucleofected Day 0 CB cells co-cultured with (+) or without (−) BMSC (xls file).** Significantly expressed gene sets were determined from normalized Illumina array data using the GSEA computational method, as described [45]. Shown are rank order lists of GSEA results from day 3 (+/− BMSC-primed) CB microarray samples, as described in Methods. These curated sets of genes were analyzed using GSEAP v2.07 (http://www.broad.mit.edu/gsea) software and the MSigDB v. 3.0 CP (canonical pathways) gene sets, with an FDR <0.05, and nominal p value<0.05 were chosen as thresholds for significance. The GSEA method determined that several distinct pathways were significantly over-represented in +BMSC-primed CB cells (+BMSC CB > –BMSC CB). These pathways included highly significant enrichment foractivation of CB cells by 1) GF and contact-dependent pathways that signal through JAK-STAT3 and are downstream of Toll Receptor-NFκB activation, 2) NFkB signaling pathways that suppress apoptosis, and 3) pathways that activate WNT signaling, destabilize p53, and regulate cell cycle transition
(XLS)
