# Supplementary figures and images for: Growth Factor-Activated Stem Cell Circuits and Stromal Signals Cooperatively Accelerate Non-Integrated iPSC Reprogramming of Human Myeloid Progenitors
Source: PLoS One. 2012 Aug 8;7(8):e42838. doi: 10.1371/journal.pone.0042838 (PMC3414503; doi:10.1371/journal.pone.0042838)

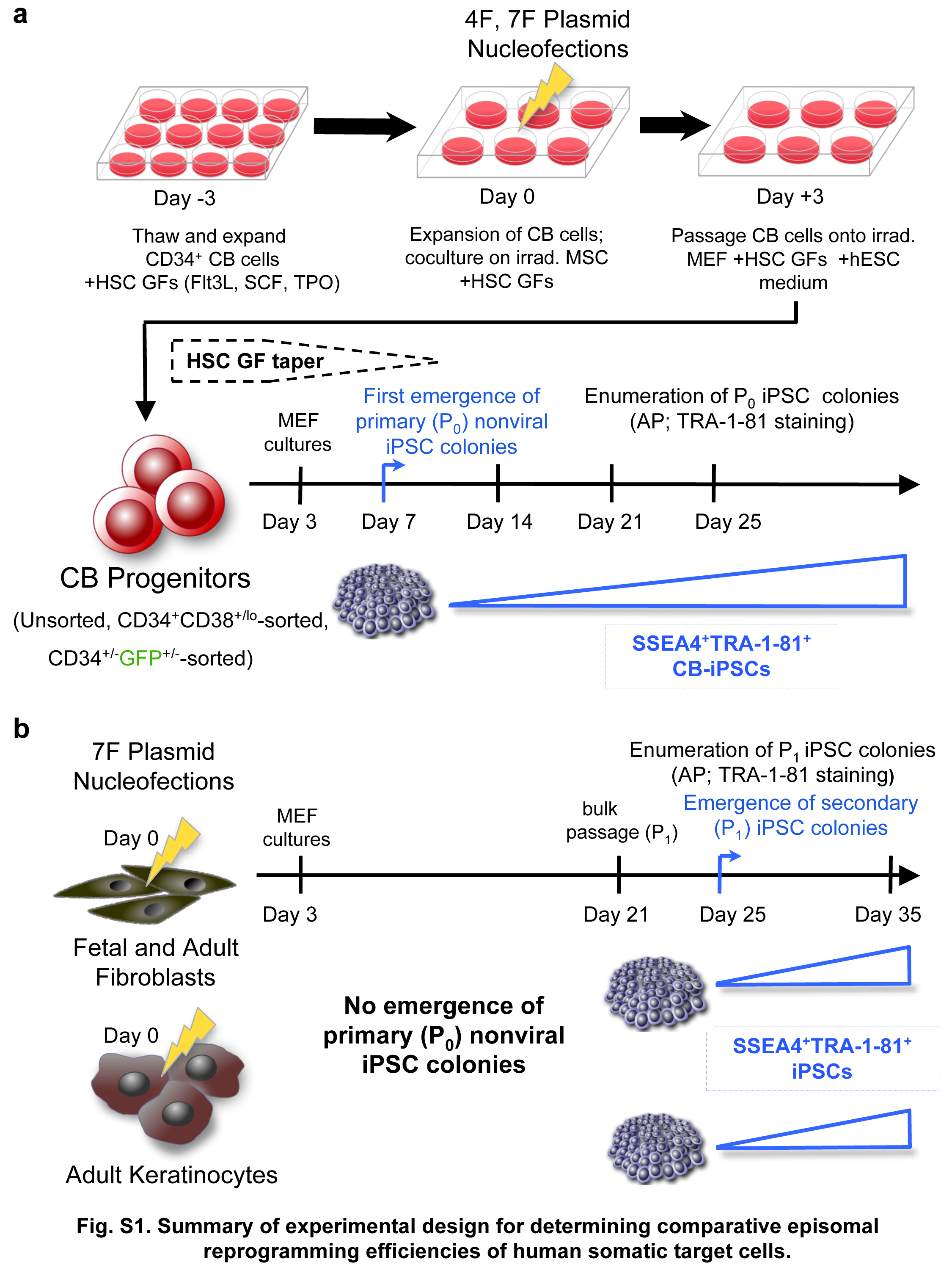

Supplement: Figure S1 — Summary of experimental design for determining comparative episomal reprogramming efficiencies of human somatic target cells. Details of reprogramming efficiency assays are described in Methods. Reprogramming efficiencies of (a) GF-activated and +/− BMSC-primed CB progenitors, or (b) fetal and adult fibroblast (FFB; AdFib) and adult keratinocyte (Ker) populations were determined on MEF cultures (plated on day +3) following plasmid nucleofections on Day 0 with a single plasmid expressing four (4F) transgenes, or three plasmids expressing seven (7F) episomal transgenes. Day 0 nucleofected CB cells were briefly co-cultured with (or without) irradiated adult BMSC stromal layers and continued hematopoietic GFs (Flt3L, TPO, Kit ligand-SCF (FTK)) from Day 0 to Day +3. Single cells were subsequently re-plated on irradiated MEF and cultured in hESC medium supplemented with 40 ng/ml bFGF. Reprogramming efficiencies of emerging hESC-like (CB-iPSC) colonies were determined in these original (P0) MEF cultures at 3–5 weeks post initial nucleofections. After 12 days on MEF, cultures were fed daily with MEF-conditioned medium (CM) supplemented with 40 ng/ml bFGF. Reprogramming efficiencies for somatic targets were determined via two independent methods in averaged triplicate-quadruplicate cultures for each experiment: 1) counting the number of colonies that emerged per single cells plated on replicate P0 MEF cultures at day 21–25 that had hESC-like morphology (as defined by compact hESC characteristics with large nuclei and nucleoli and that had high alkaline phosphatase staining (AP+; AlkPhoshi). Alternatively, 2) hESC-like colonies, as defined above, and that were positive for live Tra-1-81 surface staining were enumerated in replicate cultures. In many experiments, both assays were done in parallel on the same cultures (live TRA staining, followed by fixation and AP stating (see Fig. S2). hESC-like/AP+/Tra-1-81+ colonies emerged from nucleofected CB as early as 7–21 days post-nu [file pone.0042838.s001.tif]

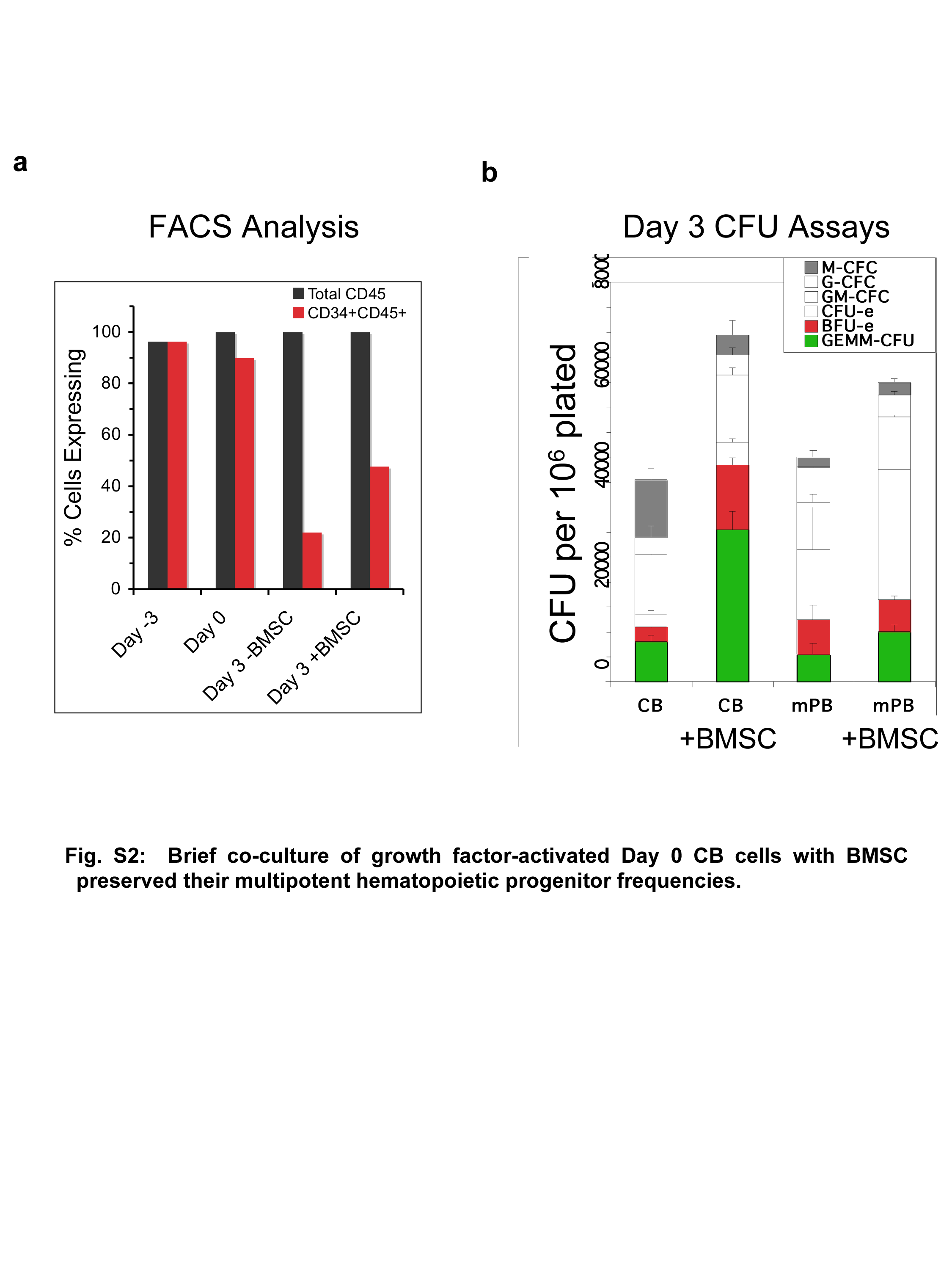

Supplement: Figure S2 — Brief co-culture of growth factor-activated Day 0 CB cells with BMSC preserved their multipotent hematopoietic progenitor frequencies. Brief co-culture of GF-activated CB cells (Flt3L, SCF, and TPO from Day -3 to Day 3) with irradiated BMSC plus continued GFs for an additional 3 days (from Day 0 to Day 3 of reprogramming protocol; see Fig. S1) increased the frequency of a) Day 3 phenotypic multipotent hematopoietic CD34+CD45+ progenitors and b) Day 3 erythro-myeloid GEMM-CFU. There was enhancement to a lesser extent in mobilized CD34+ peripheral blood progenitors (mPB). CFU colony assays of GF-activated Day 0 CB cells were conducted in semi-solid methylcellulose as previously described [54], [55]. (TIF) [file pone.0042838.s002.tif]

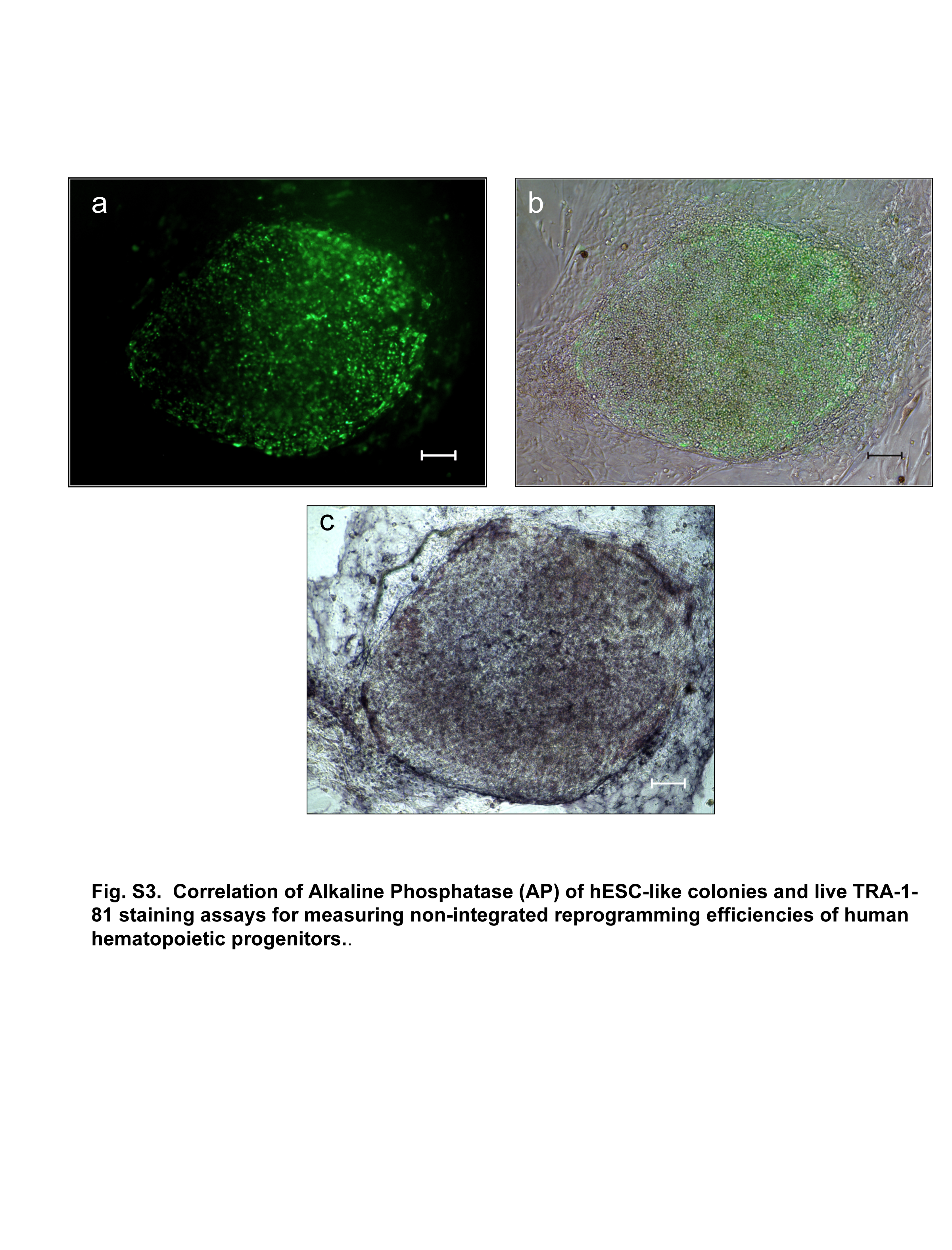

Supplement: Figure S3 — Correlation of Alkaline Phosphatase (AP) of hESC-like colonies and live TRA-1-81 staining assays for measuring non-integrated reprogramming efficiencies of human hematopoietic progenitors. Efficiencies for the number of hESC-like colonies that emerged per single cells input into Day 3 MEF cultures was determined in parallel 3–5 weeks following 4F and 7F nucleofections with two independent methods: 1) AP+ staining and 2) live TRA-1-81 staining (Dylight 488, green). Details of assays are described in Methods. Shown here are photomicrographs of a representative early P0 hESC-like colony which emerged 14 days following MEF plating of single 4F-nucleofected Day 0 CB progenitor cells, as described in text. (a) hESC-like colony after live TRA-1-81 immunostaining, (b) Same ESC-like colony, with merged phase contrast and live immunofluorescent TRA-1-81 staining. (c) Same hESC-like colony following fixation and AP staining (purple). Note that live TRA-1-81 staining (which indicates conversion to a completed Type III reprogrammed state) is more heterogeneous in early P0 colonies, and appeared with slower kinetics than the rapid dark AP staining which was dark and homogenously stained. Scale bars = 100 µ (microns). (TIF) [file pone.0042838.s003.tif]

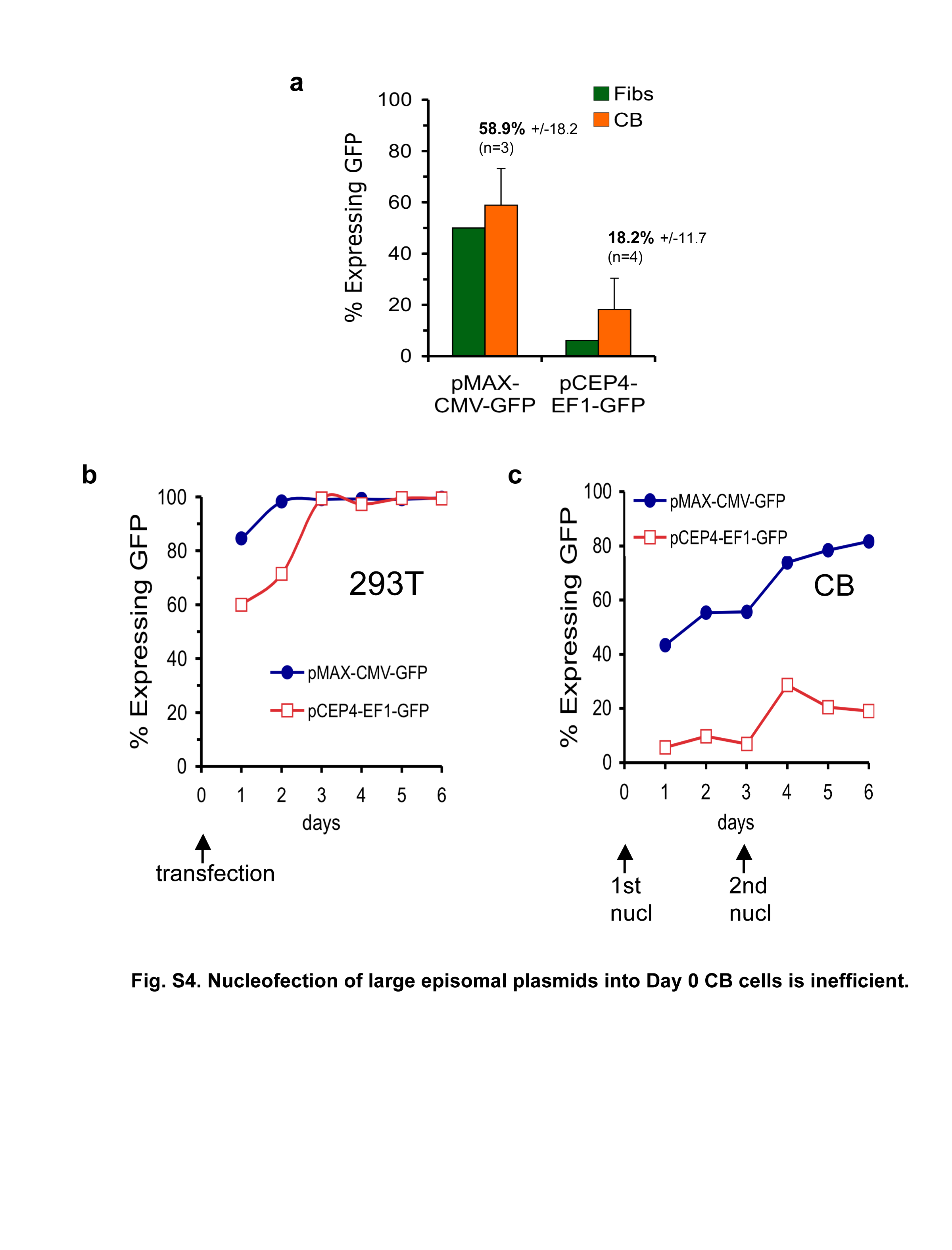

Supplement: Figure S4 — Nucleofection of large episomal plasmids into Day 0 CB cells is inefficient. Gene transfer efficiency of Day 0 CB progenitors, adult human fibroblasts, or 293T embryonic kidney carcinoma cells was determined by GFP reporter expression with either a 3 kb CMV-GFP plasmid (pMAX-CMV-GFP; AMAXA kit) or a ∼15 kb EBNA-based GFP episome (pCEP4-EF1-GFP) of similar size, and with the same promoter and vector backbone as our 4F and 7F reprogramming plasmids. (a) Results of averaged experiments for 48 hr GFP expression of Fibs or CB cells nucleofected on Day 0 with 6 µg plasmids per 500,000 cells. Time courses of GFP expression following (b) 293T transfections (Lipofectamine 2000), or (c) BMSC-primed CB nucleofections of each indicated plasmid. These experiments revealed that large pCEP4 EBNA-based episomes were excellent expression vectors via transfection, but possessed limiting nucleofection gene transfer efficiency, likely due to their large sizes. A second pulse of plasmid (c, 2nd nucl) was nucleofected on day 3 in some experiments, but did not dramatically improve the low gene transfer efficiency of the original pulse (1st nucl). (TIF) [file pone.0042838.s004.tif]

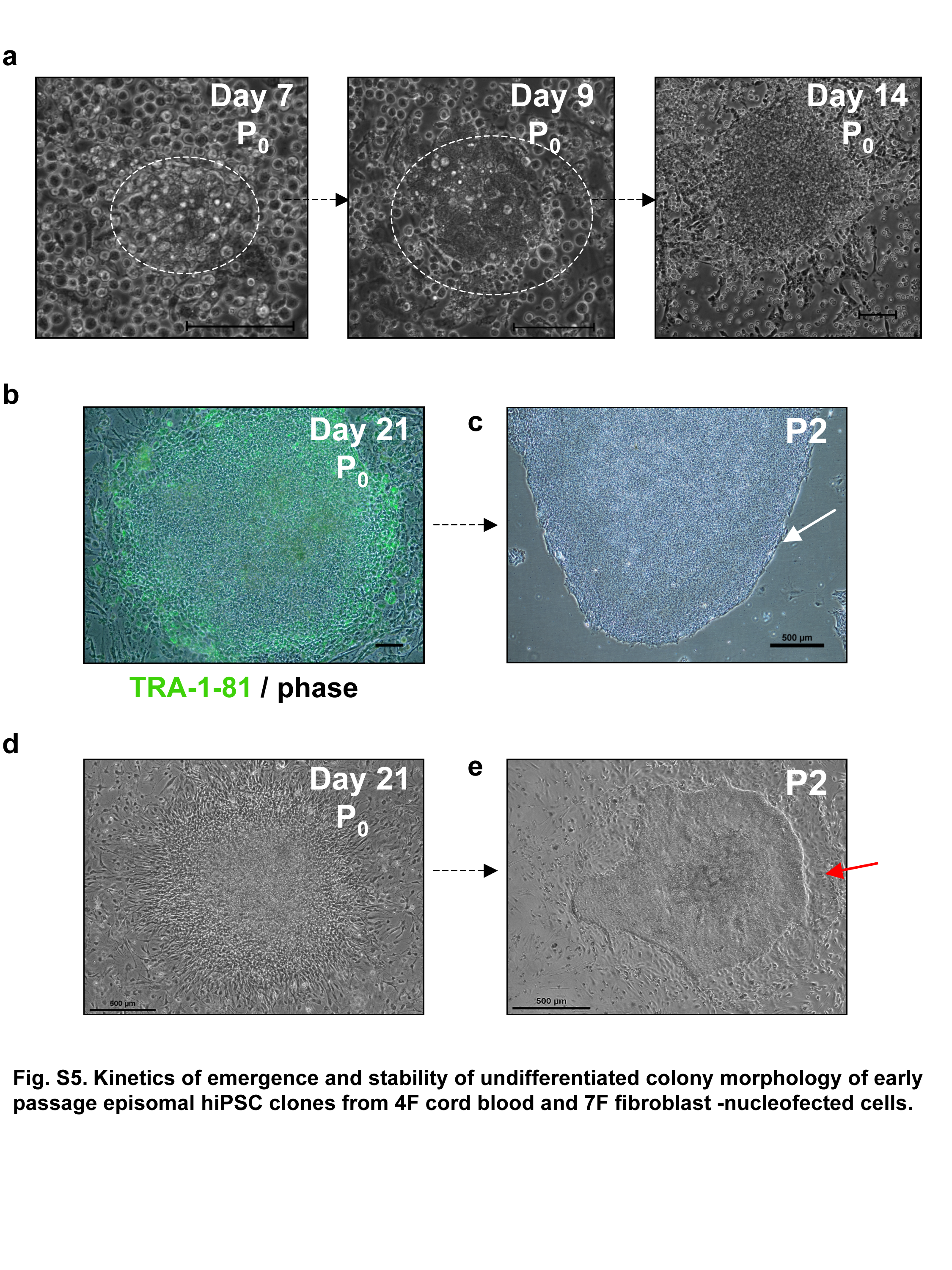

Supplement: Figure S5 — Kinetics of emergence and stability of undifferentiated colony morphology of early passage episomal hiPSC clones from 4F cord blood and 7F fibroblast -nucleofected cells. TRA-1-81+ hESC-like colonies emerged rapidly with 4F-nucleofected BMSC-primed CD34+ CB cells (as early as 7–14 days in initial P0 MEF co-cultures), and at significantly higher efficiencies than without BMSC co-culture. Additionally, unlike episomal fetal fibroblast-iPSC or non-BMSC-primed CB-iPSC colonies (not shown), the majority (>90%) of BMSC-primed CB-iPSC clones maintained a stable undifferentiated hESC-like morphology with minimal spontaneous differentiation that permitted manual picking and expansion with minimal effort. Shown in (a) is typical morphology of an emerging 4F CB-iPSC clone at day 7, 9,14 and 21 with (b) live TRA-1-81 staining), as well as following expansion at (c) passage 2 (P2). Note the stable, well-circumscribed borders of the P2 episomal CB-iPSC colony with minimal differentiation (white arrow). In contrast, although abnormal granulated colonies (d) often emerged for 7F-nucleofected fibroblasts at P0, colonies with hESC-like morphology did not, and only emerged with slow kinetics after 1–2 MEF passages, and with unstable spontaneous differentiation that required extensive subcloning. Shown in (e) is a representative 7F fib-iPSC colony at P2 with typical spontaneously differentiating borders (red arrow). (TIF) [file pone.0042838.s005.tif]

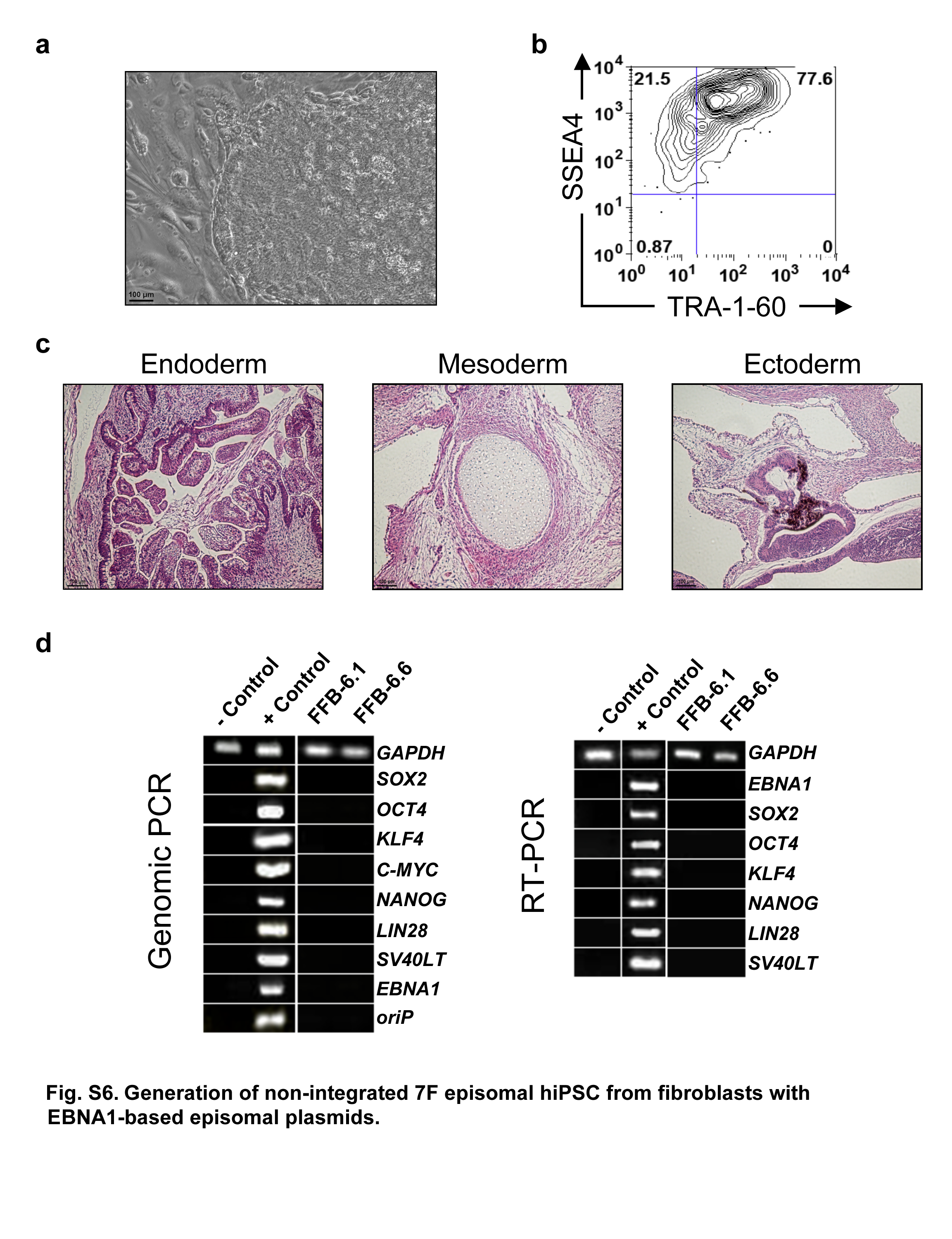

Supplement: Figure S6 — Generation of non-integrated 7F episomal hiPSC from fibroblasts with EBNA1-based episomal plasmids. 22 week-old lung fetal fibroblasts carrying the homozygous sickle cell disease mutation were obtained from the Coriell Cell Repository (GM02340), and used to generate nonviral human fetal fibroblast-derived SSEA4+TRA-1-60+ hiPSC (a,b; at low passage) with seven episomal factors, as described in the text, that (c) demonstrated differentiation to all three germ layers in NOG teratoma assays. Shown are H&E stains of teratoma sections from SCD-hiPSC demonstrating elements of ectoderm (neural rosettes, retinal pigmented epithelium, endoderm (glandular epithelium), and mesoderm (bone, muscle). Shown also (d) are genomic PCR and RT-PCR assays confirming the lack of integration and expression of transgenic episomal constructs. Details for these transgene-specific PCRs were previously described [5]. (TIF) [file pone.0042838.s006.tif]

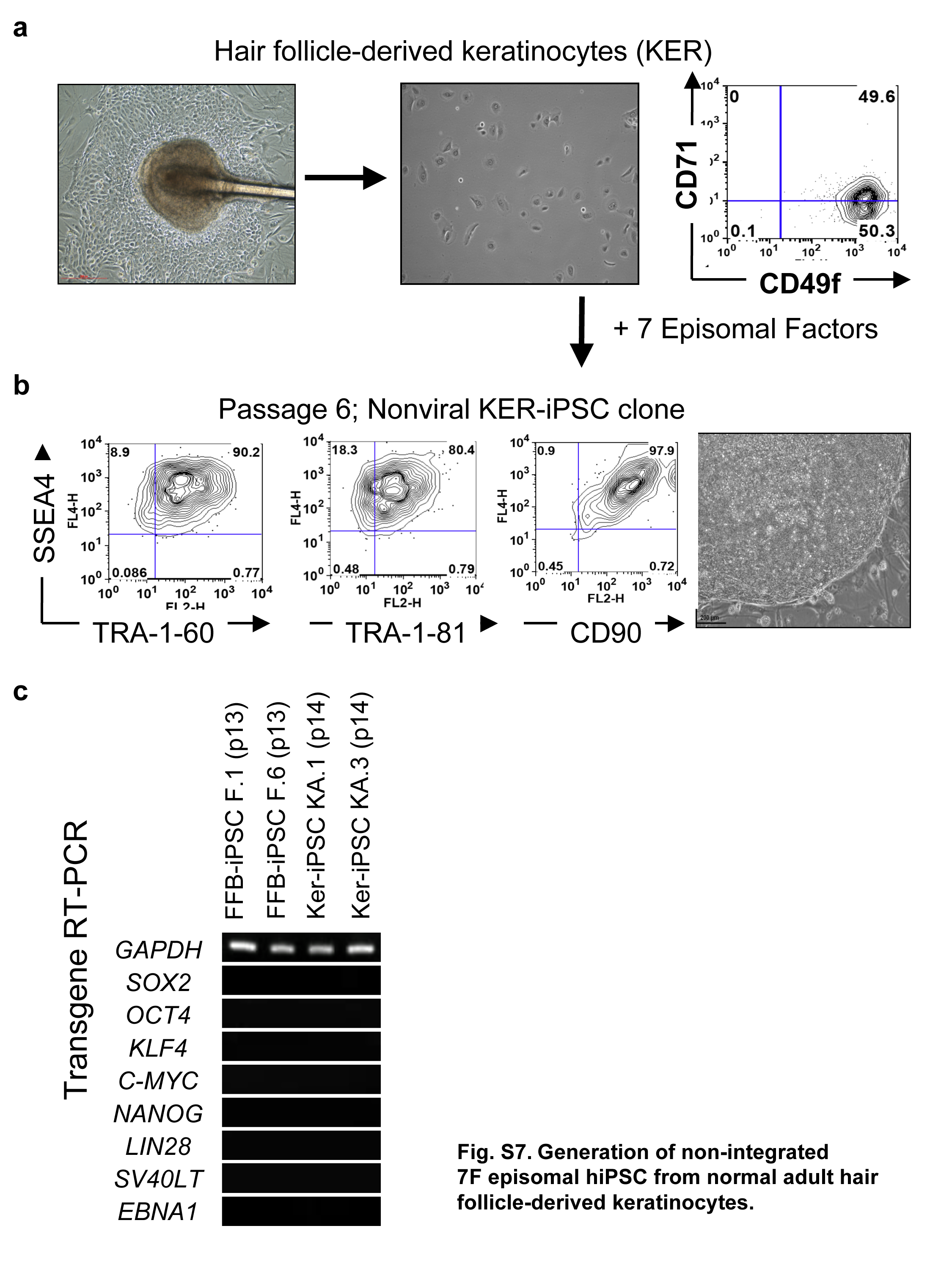

Supplement: Figure S7 — Generation of non-integrated 7F episomal hiPSC from normal adult hair follicle-derived keratinocytes. Keratinocyte lineage cells were confirmed by CD49f (alpha-integrin)-positive, CD71-low cells after expansion from a single plucked hair (a; left panel) of a normal adult donor, using methods as described [53]. ∼2×10^6^ cell were nucleofected with “Combo 6″, re-suspended in fresh culture medium, and then transferred onto gelatinized PMEF plates. After 48–72 hours, media was replaced with hESC medium or CM for three weeks (P0), followed by replating onto fresh PMEF (P1). (b) Colonies with hESC-like morphology, and expressing pluripotency markers (e.g., SSEA4, Tra-1-60/81, CD90, OCT4, NANOG, SOX2), emerged with rare efficiencies (see Fig. 1 ) 1–2 weeks following P1 culture of nucleofected cells. Nonviral iPSC clones derived from keratinocytes. KER-iPSC were further subcloned, and confirmed for lack of integrated episomal sequences by RT-PCR (c) of pluripotency transgenes, expanded for frozen stocks, and confirmed for pluripotency by tri-lineage cystic teratoma formation assay (not shown). (TIF) [file pone.0042838.s007.tif]

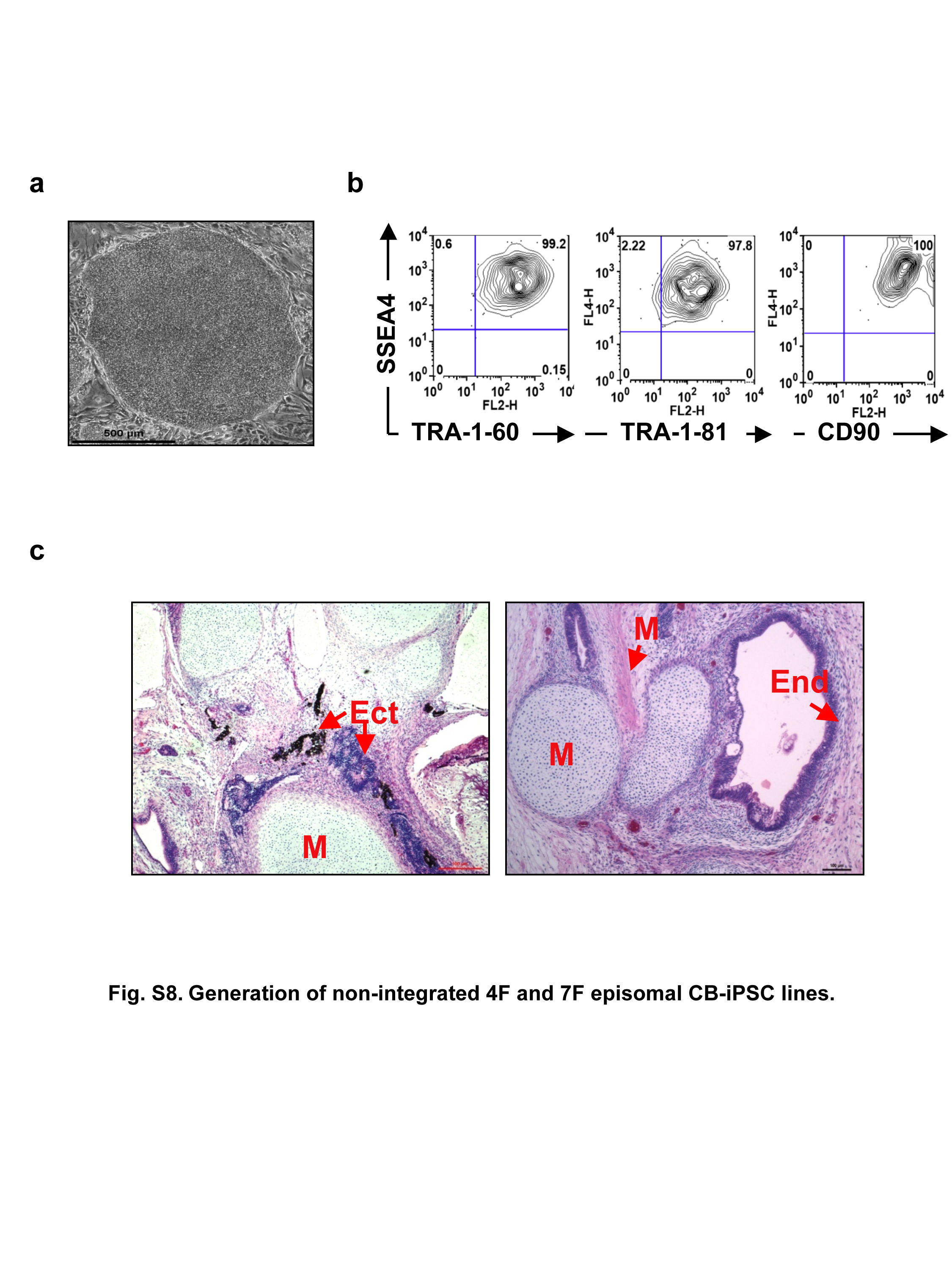

Supplement: Figure S8 — Generation of non-integrated 4F and 7F episomal CB-iPSC lines. (a,b) Representative colony morphology (a) and FACS staining (b) of SSEA4, TRA-1-81, and CD90 surface pluripotency markers from CB-iPSC lines generated as described in Methods, with four (4F) or seven (7F) episomal reprogramming factors. Full characterizations, including Southern blots, genomic PCR, and RT-PCR for validations of vector and transgene-free status of CB-iPSC lines that we evaluated by expression microarrays in Fig. S10 (i.e., clones 6.2, 6.11, 6.13, and 19.11) were previously reported [5]. (c) H&E stains of cystic teratomas obtained from a representative CB-iPSC line 6–8 weeks following injection into NOD/SCID mice illustrate well-differentiated cell lineages of all three germ layers, including regions containing neural rosettes, pigmented retinal epithelium, glandular epithelium, fetal intestinal structures, cartilage, striated muscle, and hyalinized bone. Ectodermal structures (Ect): neural rosettes (left); retinal pigmented epithelium (right); Endodermal structures (End): glandular epithelium (left); developing gut loop (right); Mesodermal structures (M): cartilage (left), bone/muscle (right). All CB-iPSC lines described in this manuscript formed similar tri-lineage cystic teratomas. Analysis of histological sections also demonstrated that these teratomas were completely devoid of foci of malignant transformation. Scale bars = 100 µ (microns). (TIF) [file pone.0042838.s008.tif]

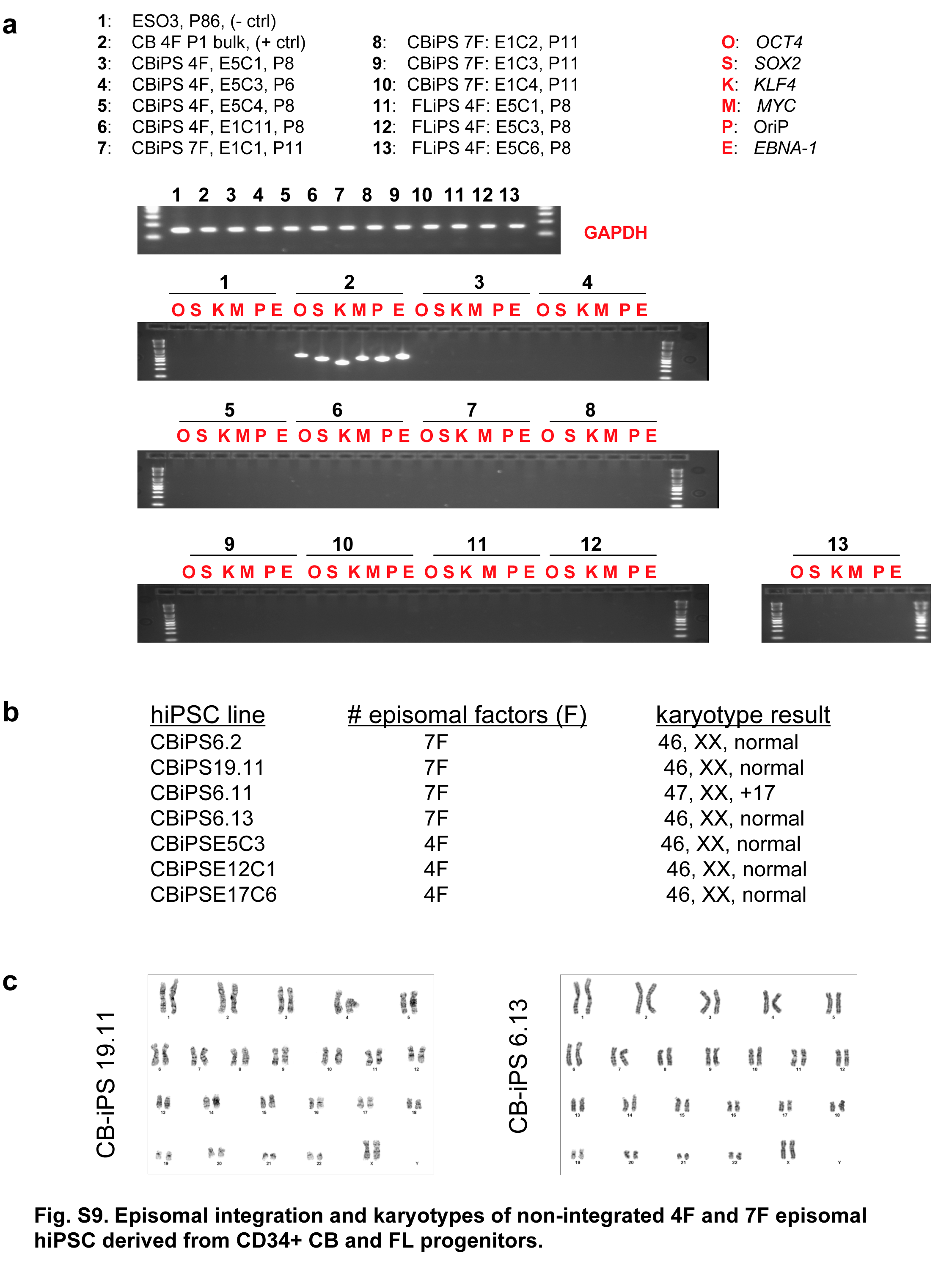

Supplement: Figure S9 — Episomal integration and karyotypes of non-integrated 4F and 7F episomal hiPSC derived from CD34+ CB and FL progenitors. (a,b) 4F and 7F CB-iPSC and FL-iPSC were assayed by transgene-specific genomic PCR at indicated passages exactly as previously described [5] for episomal sequences. Bulk P1 4F CB-iPSC cultures serve as a positive control. (c) G-band karyotyping on representative 4F and 7F CB-iPSC lines. Experimental details for genomic PCR and karyotyping are provided in Methods. (TIF) [file pone.0042838.s009.tif]

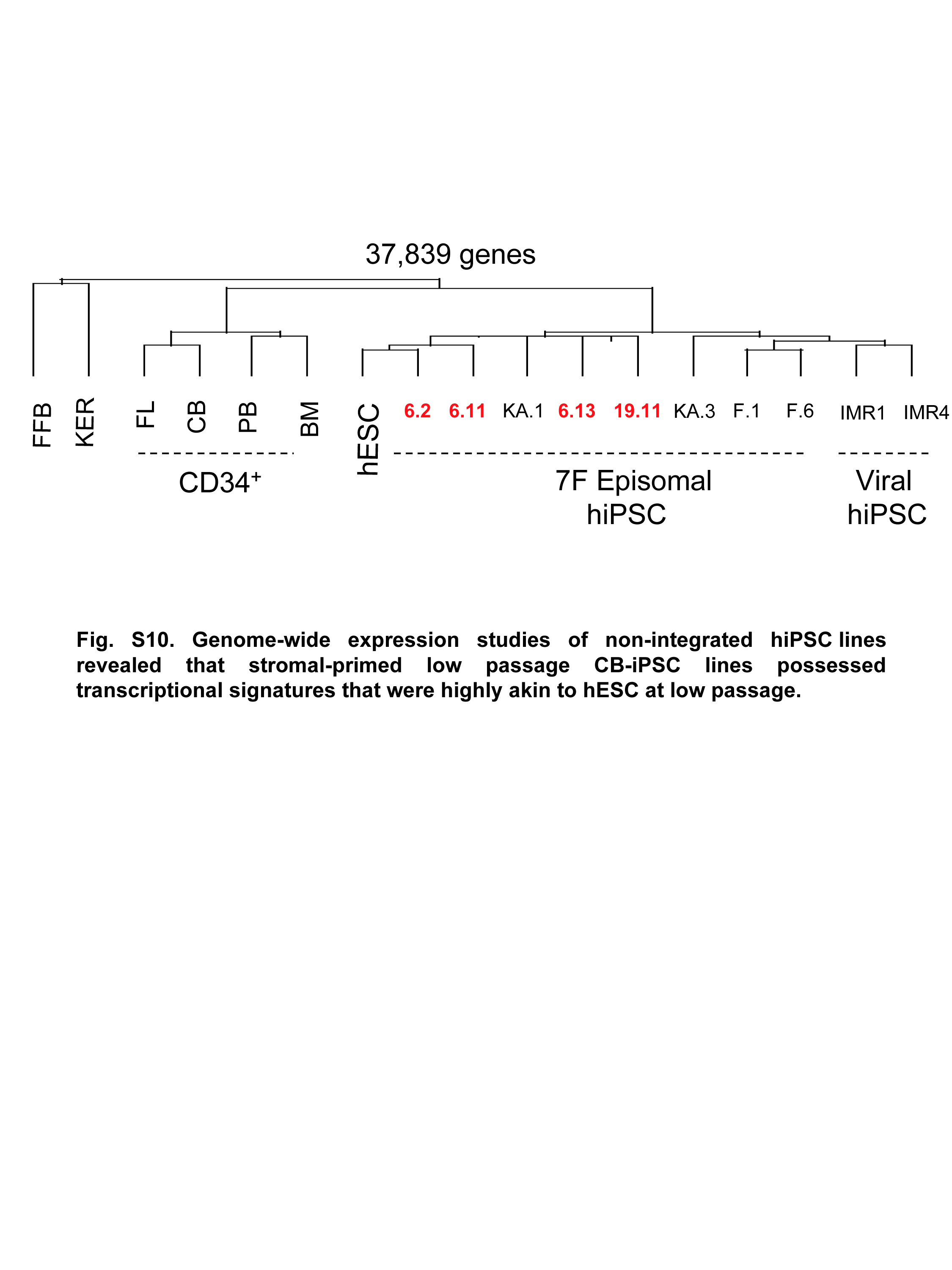

Supplement: Figure S10 — Genome-wide expression studies of non-integrated hiPSC lines revealed that stromal-primed low passage CB-iPSC lines possessed transcriptional signatures that were highly akin to hESC at low passage. To examine the quality of nonintegrated reprogramming achieved, low passage hiPSC clones were derived from fetal fibroblasts (Fig. S5), keratinocytes (Fig. S6), as well as stromal-primed CB donors (Fig. S7, S8). And their transcriptional signatures were evaluated by Illumina microarray expression analysis. Non-integrated hiPSC were generated with the same 7F episomal constructs, and global gene expressions were compared. All non-integrated hiPSC lines were confirmed to be free of transgene and vector sequences by Southern blotting, genomic PCR, and RT-PCR at early passage (p9–12), as previously described [5] and (Figs. S5, S6, S7). Levels of pluripotency markers SSEA4, TRA-1-60, TRA-1-81, OCT4, and NANOG proteins for all hiPSC assayed were found comparable to control hESC. All non-integrated iPSC lines were also tested for their ability to form, well-differentiated tri-lineage cystic teratomas in NOG-SCID mice demonstrating their bona fide pluripotency. We determined the expression signatures of these non-integrated hiPSC clones with Illumina microarrays, and also included previously described lentiviral hiPSC lines IMR90-1 and IMR90-2 [56] and H9 hESC as controls. An unsupervised hierarchical clustering of global expression (37,839 genes) from all starting populations and cell lines was computed. Global gene expression samples of episomal lines were evaluated at the earliest passage possible (P11–14). H9 hESC (P51), episomal CB-iPSC5 clones 6.2, 6.11, 6.13, (P14), 19.11, (P11), nonviral keratinocyte-iPSC clones: KA.1, KA.3 (P13); episomal fetal fibroblast-iPSC: F.1, F.6 (P14); viral fibroblast-iPSC clones: IMR1 (P66), IMR4 (P64). This dendrogram represents the unsupervised hierarchical clustering of signal values from all 37,839 genes represented on the Illumina microar [file pone.0042838.s010.tif]

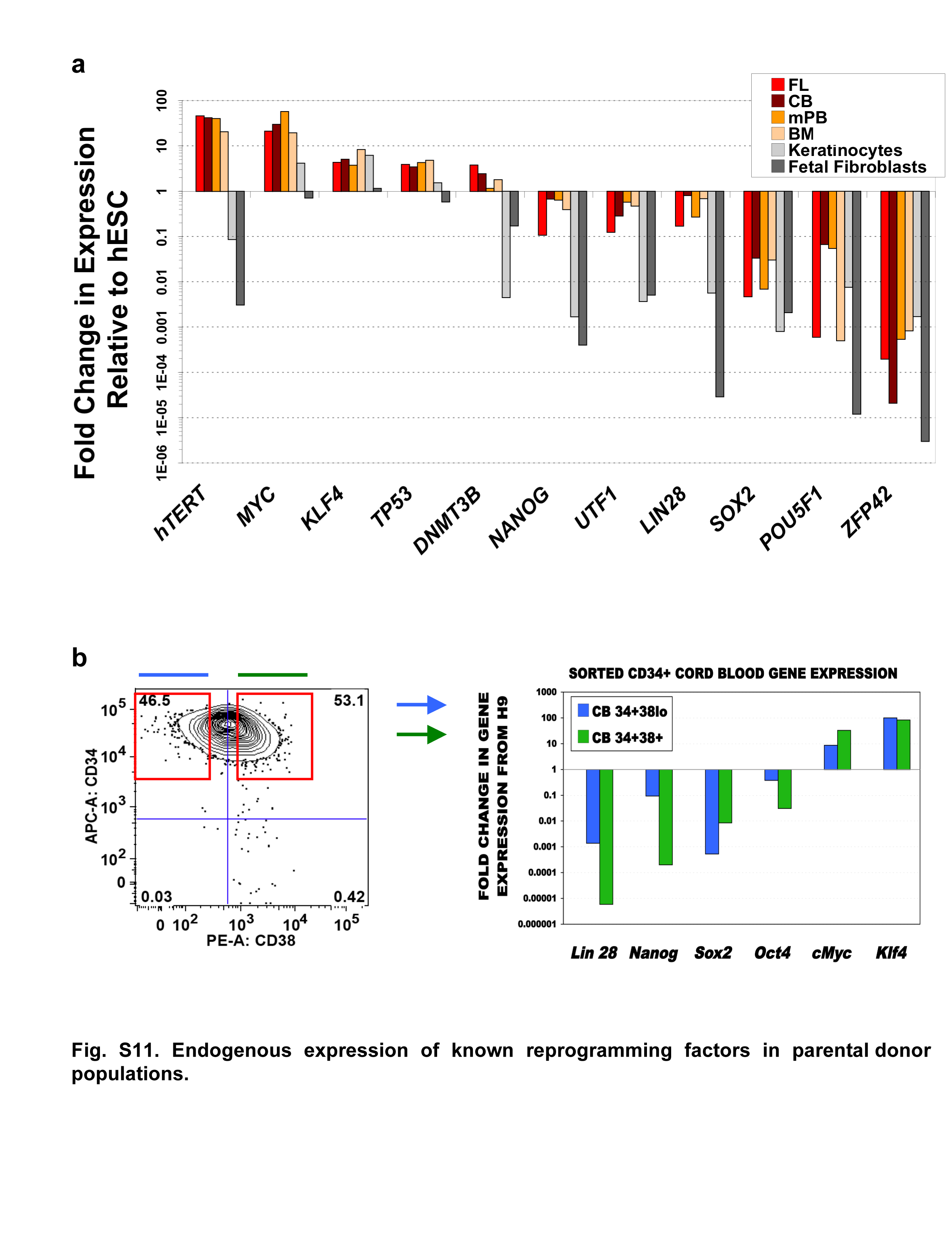

Supplement: Figure S11 — Endogenous expression of known reprogramming factors in parental donor populations. (a) Endogenous expressions of pluripotency-associated factors were determined by qRT-PCR analysis on Day 0 of the reprogramming protocol of GF-activated (FTK) donor cell populations (CD34+ fetal liver; FL), CD34+ cord blood (CB), GCSF-mobilized peripheral CD34+ blood (mPB), adult CD34+ bone marrow (BM), adult keratinocytes, and fetal fibroblasts. Shown is the fold change normalized expression levels of each factor relative to expression in control H9 hESC calculated by the 2−ΔΔCT method. Primer sequences are presented in Methods. (b) Stem-progenitor (CD34+CD38lo) and lineage-committed (CD34+CD38+) populations were FACS-purified from Day -2.5 CB cells, and similarly evaluated for expression of endogenous pluripotency factor transcripts by qRT-PCR. (TIF) [file pone.0042838.s011.tif]
